# Preparation of Complexes Bearing N‐Alkylated, Anionic or Protic CAACs Through Oxidative Addition of 2‐Halogenoindole Derivatives

**DOI:** 10.1002/anie.202010988

**Published:** 2020-11-30

**Authors:** Sebastian Termühlen, Jonas Blumenberg, Alexander Hepp, Constantin G. Daniliuc, F. Ekkehardt Hahn

**Affiliations:** ^1^ Institut für Anorganische und Analytische Chemie Westfälische Wilhelms-Universität Münster Corrensstrasse 30 48149 Münster Germany; ^2^ Organisch-Chemisches Institut Westfälische Wilhelms-Universität Münster Corrensstrasse 40 48149 Münster Germany

**Keywords:** CAAC complexes, chloro indole, chloro indolium salts, oxidative addition, protic CAACs

## Abstract

CAAC precursors 2‐chloro‐3,3‐dimethylindole **1** and 2‐chloro‐1‐ethyl‐3,3‐dimethylindolium tetrafluoroborate **2**BF_4_ have been prepared and oxidatively added to [M(PPh_3_)_4_] (M=Pd, Pt). Salt **2**BF_4_ reacts with [Pd(PPh_3_)_4_] in toluene at 25 °C over 4 days to yield complex cis‐[**3**]BF_4_ featuring an N‐ethyl substituted CAAC, two cis‐arranged phosphines and a chloro ligand. Compound trans‐[**3**]BF_4_ was obtained from the same reaction at 80 °C over 1 day. Salt **2**BF_4_ reacts with [Pt(PPh_3_)_4_] to give cis‐[**4**]BF_4_. The neutral indole derivative **1** adds oxidatively to [Pt(PPh_3_)_4_] to give trans‐[**5**] featuring a CAAC ligand with an unsubstituted ring‐nitrogen atom. This nitrogen atom has been protonated with py⋅HBF_4_ to give trans‐[**6**]BF_4_ bearing a protic CAAC ligand. The Pd^II^ complex trans‐[**7**]BF_4_ bearing a protic CAAC ligand was obtained in a one‐pot reaction from **1** and [Pd(PPh_3_)_4_] in the presence of py⋅HBF_4_.

## Graphical abstract

Cyclic (alkyl)(amino)carbene (CAAC) precursor salt **2**BF_4_ reacts with [M(PPh_3_)_4_] (M=Pd, Pt) to give *cis/trans*‐[**3**]BF_4_ and *cis*‐[**4**]BF_4_, in which the CAAC ligand bears an unusual N‐alkyl substituent. *trans*‐[**5**] has a unique anionic CAAC ligand with an unsubstituted ring‐nitrogen atom which can be protonated to give *trans*‐[**6**]BF_4_ bearing a protic CAAC (*p*CAAC) ligand.

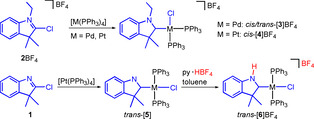

The preparation of cyclic (alkyl)(amino)carbenes (CAACs, **A** in Figure 1) by Bertrand in 2005^[1]^ added an interesting new derivative to the large family of N‐heterocyclic carbenes (NHCs, **B** in Figure 1).^[2]^ Over the last years, various applications for CAAC ligands in transition metal coordination chemistry, catalysis and for the stabilization of reactive species have been described.^[3]^ Compared to the ubiquitous N‐heterocyclic carbenes, CAACs feature special electronic properties,^[3e]^ which also enable the activation of small molecules such as CO,^[4]^ P_4_ ^[5]^ and even H_2_ or NH_3_.^[6]^

**Figure 1:**
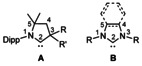
CAACs **A** and NHCs **B**.

Generally, CAACs of type **A** are obtained by C2‐deprotonation of cyclic aldiminium salts. Such salts are available from the reaction of aldimines with epoxides^[1]^ or 3‐halogeno‐2‐methylpropenes^[7]^ followed by acid catalyzed cyclization. While these routes have led to cyclic aldiminium salts and CAACs bearing various R and R′ groups at the quaternary carbon atom C3, the choice of the amino substituent is restricted to aromatic, bulky, electron‐withdrawing groups such as 2,6‐diisopropylphenyl. In addition, the sp^3^‐carbon atom C5 must be quaternary as C2‐deprotonation of the aldiminium cation would otherwise compete with deprotonation at C5. The replacement of one electronegative and planar π‐donating amino group in NHCs **B** for a tetrahedral σ‐ but not π‐donating alkyl group in the CAACs **A** make the latter ones both more nucleophilic (σ‐donating) and more electrophilic (π‐accepting). Computational studies show that the HOMO of CAACs lies slightly higher in energy than in NHCs and the singlet‐triplet energy gap in CAACs is slightly smaller than in NHCs.^[6]^ CAAC complexes are normally prepared from the (in situ generated) free CAAC ligands and suitable metal complexes.

We became interested in the preparation of the currently unknown complexes bearing CAACs with aliphatic N1‐substituents or even a proton at N1. Given the instability of free CAACs in the absence of bulky aromatic substituents at N1, we assumed that the target N‐alkyl CAACs will not be stable in the free state and that complex preparation must therefore proceed via the in situ generation of the CAAC from a suitable precursor.

Various NHC complexes have been prepared by the in situ deprotonation of azolium salts in the presence of suitable metal complexes. Alternatively, but also without isolation of the free carbene, NHC complexes are accessible by the oxidative addition of the C2‐X bond (X=H, R, halogen) of azoles, azolium or pyrazolium cations to low valent transition metals.^[8]^ The oxidative addition methodology has also been employed for the preparation of NHC complexes bearing the freely unstable protic NHCs (*p*NHCs)^[9]^ or protic mesoionic NHCs.^[10]^

Encouraged by the versatility of the oxidative addition methodology, we prepared the halogenoindole derived, annulated CAAC precursors **1** ^[11]^ and **2**BF_4_ ^[12]^ (Scheme 1, see the SI) for the preparation of CAAC complexes by an oxidative addition of the C2‐Cl bond to low‐valent transition metals.

**Scheme 1:**
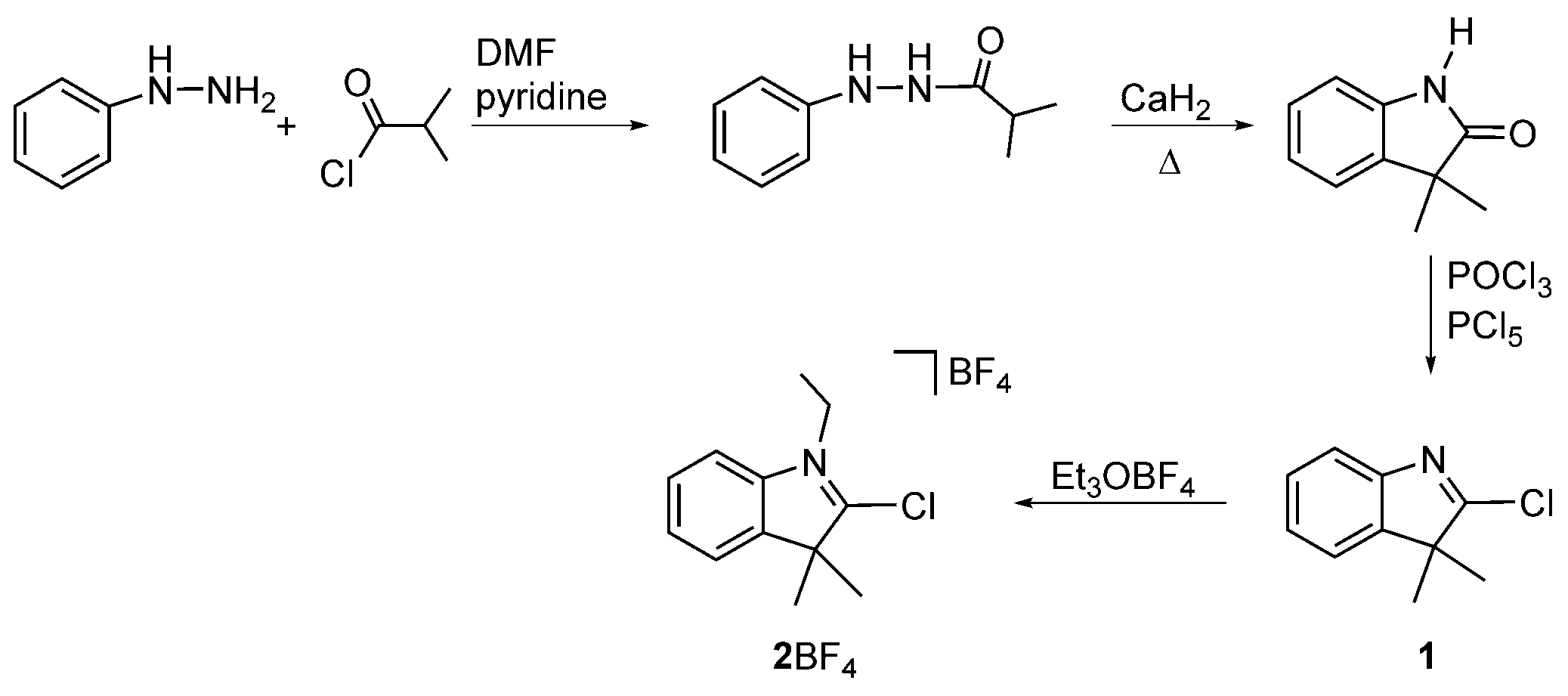
Synthesis of CAAC precursors **1** and **2**BF_4_.

Reaction of the 2‐chlorindolium salt **2**BF_4_ with one equivalent of [Pd(PPh_3_)_4_] yielded the CAAC complex *cis*‐[**3**]BF_4_ in good yield (Scheme 2, top). The heterocyclic ligand features all characteristics of the known CAAC ligands of type **A** except for the C4‐C5 annulation and the unique N1‐alkyl substitution. Typical CAAC ligand properties were also observed in the ^13^C NMR spectrum of *cis*‐[**3**]BF_4_ featuring the downfield shifted resonance for the C_CAAC_ carbon atom at *δ*(C2)=242.2 (*cf* the C_NHC_ resonance at *δ*(C2)≈165–175 found in related NHC‐Pd complexes.[^9a^, ^9d^, ^9e^]). The observation of a doublet of doublets for C2 (^2^ *J*_CP,*trans*_=141.9 Hz, ^2^ *J*_CP,*cis*_=5.7 Hz) indicates the *cis*‐disposition of the phosphine donors in *cis*‐[**3**]BF_4_. This *cis*‐arrangement was confirmed by the observation of two resonances in the ^31^P NMR spectrum at *δ*=29.8 (^2^ *J*_PP_=26.0 Hz, P_*trans*_) and *δ*=19.6 (^2^ *J*_PP_=26.0 Hz, P_*cis*_). Some related complexes obtained from isoindolium salts (featuring cyclic (amino)(aryl)carbenes, CAArC)[^7^, ^13^] or indazolium salts^[14]^ have been described. However, these carbene ligands differ significantly from **A** or the CAAC ligand in *cis*‐[**3**]BF_4_ by the aromatic character of the carbon atom C3 which in both cases is part of a C3‐C4 annulated benzene ring.

**Scheme 2:**
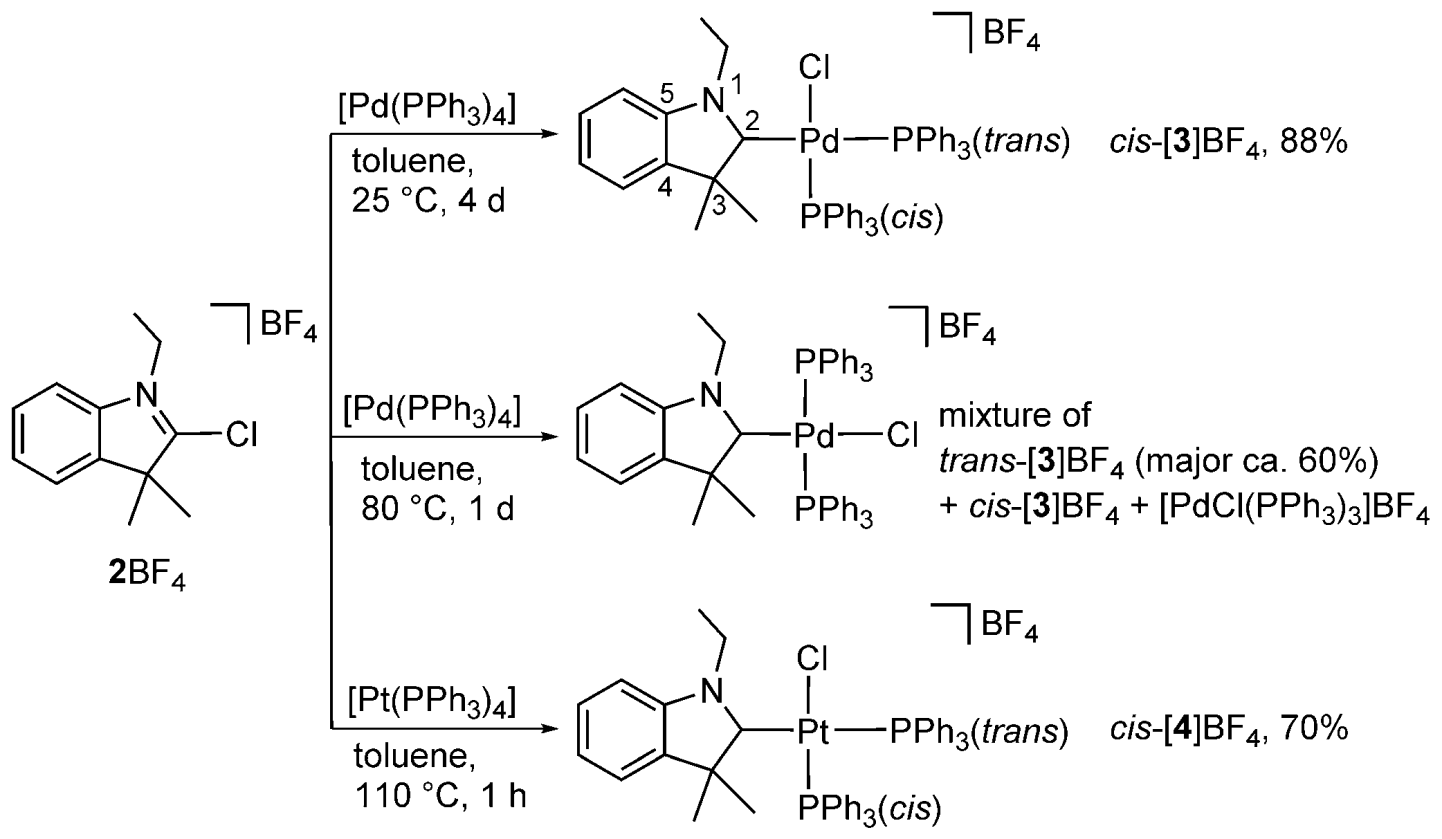
Oxidative Addition of **2**BF_4_ to [M(PPh_3_)_4_].

The oxidative addition of **2**BF_4_ to [Pd(PPh_3_)_4_] at an elevated temperature of 80 °C yields a complex mixture composed of *trans*‐[**3**]BF_4_ (major, about 60 %), *cis*‐[**3**]BF_4_ and [PdCl(PPh_3_)_3_]BF_4_ (Scheme 2, middle). While the components of the complex mixture could not be separated, the individual complexes in the mixture were identified by ^31^P NMR spectroscopy (see the SI). Complexes *cis*‐[**3**]BF_4_ and [PdCl(PPh_3_)_3_]BF_4_ were identified by comparison with the ^31^P NMR spectra of authentical samples while *trans*‐[**3**]BF_4_ gave the expected singulet resonance at *δ*=24.2. The temperature dependent formation of *cis*‐ and *trans*‐isomers has been previously observed during the oxidative addition of halogenoazoles to [M(PPh_3_)_4_] (M=Pd, Pt) complexes^[9a]^ confirming that the CAAC ligands behave similarly to normal NHC ligands. Related observations have been made for palladium(II) complexes of type [Pd(*r*NHC)(PPh_3_)_2_I] (*r*NHC=pyrazolin‐4‐ylidene), where the initially formed *cis*‐diphosphine complex slowly transforms into the thermodynamically more stable *trans*‐complex.^[9f]^ The preference for the *trans*‐complex has been rationalized by the transphobia effect, a term proposed for the difficulty of placing a phosphine donor *trans* to a carbon donor.^[9g]^

Finally, the oxidative addition of **2**BF_4_ to [Pt(PPh_3_)_4_] yielded complex *cis*‐[**4**]BF_4_ (Scheme 2, bottom). Even at the elevated temperature of 110 °C, only the *cis*‐complex cation was observed in accord with the enhanced kinetic inertness of platinum(II) complexes.^[9b]^ However, the reaction was completed after only 1 h and prolonged heating might also lead to the thermodynamically favored *trans*‐complex cation.^[9a]^

Compound *cis*‐[**4**]BF_4_ was characterized by NMR spectroscopy and by mass spectrometry (see the SI). The ^13^C NMR spectrum exhibits the resonance for the carbene carbon atom at *δ*(C2)=232.9 as the expected doublet of doublets (^2^ *J*_CP*trans*_=126.7 and ^2^ *J*_CPc*is*_=8.2 Hz) for a *cis*‐diphosphine complex. The ^31^P NMR spectrum features two resonances at *δ*=15.5 (d, ^2^ *J*_PP_=20.4 Hz; d, ^1^ *J*_PPt_=2020 Hz, Pt satellites, P_*trans*_) and *δ*=11.1 (d, ^2^ *J*_PP_=20.4 Hz; d, ^1^ *J*_PPt_=3773 Hz, Pt satellites, P_*cis*_) for the two chemically different phosphorus atoms. The HRMS spectrum (ESI, positive ions) shows the strongest peak at *m*/*z=*928.2363 (calcd for [**4**]^+^=928.2369).

The molecular structures of *cis*‐[**3**]BF_4_, *trans*‐[**3**]BF_4_⋅CH_2_Cl_2_ (Figure 2) and of *cis*‐[**4**]BF_4_ (see the SI, Figure S1)^[16]^ have been determined by X‐ray diffraction, confirming the composition and coordination geometry of the complex cations. The metal atoms in the complexes are surrounded in a slightly distorted square‐planar fashion with the CAAC plane oriented almost perpendicular to the palladium coordination plane in all three cases.

**Figure 2:**
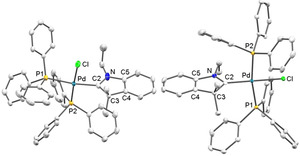
Molecular structures of complex cation *cis*‐[**3**]^+^ in *cis*‐[**3**]BF_4_ (left) and *trans*‐[**3**]^+^ in *trans*‐[**3**]BF_4_⋅CH_2_Cl_2_ (right). Hydrogen atoms have been omitted for clarity. Thermal ellipsoids are set at 50 % probability.^[16]^ Selected bond lengths [Å] and angles (°) for *cis*‐[**3**]^+^ [*trans*‐[**3**]^+^]: Pd‐Cl 2.3374(7) [2.3512(9)], Pd‐P1 2.3956(7) [2.3721(10)], Pd‐P2 2.2838(7) [2.3591(9)], Pd‐C2 2.022(3) [1.996(3)], N‐C2 1.305(4) [1.310(5)], N‐C5 1.439(4) [1.446(5)], C2‐C3 1.518(4) [1.533(5)]; Cl‐Pd‐P1 84.12(2) [84.13(3)], Cl‐Pd‐P2 177.39(3) [85.28(3)], Cl‐Pd‐C2 84.85(7) [179.61(11)], P1‐Pd‐P2 98.48(2) [168.75(4)], P1‐Pd‐C2 168.95(7) [95.83(10)], P2‐Pd‐C2 92.56(7) [94.79(10)], C2‐N‐C5 113.4(2) [112.5(3)], N‐C2‐C3 108.3(2) [108.7(3)].

The Pd‐C2 bond distance in *trans*‐[**3**]^+^ is significantly shorter than in *cis*‐[**3**]^+^ which is attributable to the weaker *trans*‐effect of the chloride ligand in *trans*‐[**3**]^+^ compared to the phosphine ligand in *cis*‐[**3**]^+^. Related observations were made for the Pd‐Cl bond distances where a short separation of 2.3374(7) Å was observed for *cis*‐[**3**]^+^ and a longer one of 2.3512(9) Å for *trans*‐[**3**]^+^. As expected, the CAAC donor exerts a stronger *trans*‐influence than the phosphine ligand. Due to the different ligand arrangements, the Pd‐P separations in *cis*‐[**3**]^+^ differ by about 0.112 Å while this difference amounts to only 0.013 Å in *trans*‐[**3**]^+^.

The metric parameters of the CAAC ligands are identical within experimental error in *cis*‐[**3**]^+^ and *trans*‐[**3**]^+^ and compare well to equivalent parameters observed in palladium complexes bearing non‐annulated CAAC ligands.^[1]^ These include a C2‐C3 single bond (1.518(4) Å in *cis*‐[**3**]^+^ and 1.533(5) Å in *trans*‐[**3**]^+^), a significantly shorter N‐C2 bond (1.305(4) Å in *cis*‐[**3**]^+^ and 1.310(5) Å in *trans*‐[**3**]^+^) and N‐C2‐C3 angles of 108.3(2)° in *cis*‐[**3**]^+^ and 108.7(3)° in *trans*‐[**3**]^+^. Comparable metric parameters for *cis*‐[**4**]^+^ are very similar to those found for *cis*‐[**3**]^+^ (see the SI, Figure S1).

Apart from azolium cations,[^8b^, ^8d^] neutral halogenoazoles can also oxidatively add to low‐valent transition metals to yield complexes bearing anionic azolato ligands,[^9a^, ^9b^, ^9c^, ^9d^, ^9e^] which after N‐protonation yield complexes bearing neutral protic NHC (*p*NHC)[^9a^, ^9c^, ^9d^, ^9e^] ligands.^[15]^ In order to prepare the analogous but currently unknown complexes bearing N1‐protonated CAAC ligands, neutral 2‐chloroindole **1** was reacted with [Pt(PPh_3_)_4_] to give complex *trans*‐[**5**] bearing an anionic CAAC ligand with an unsubstituted ring‐nitrogen atom (Scheme 3, top). The *trans*‐disposition of the phosphine ligands in this case most likely results from the extended reaction time of 1 d (compared to only 1 h for the synthesis of complex *cis*‐[**4**]BF_4_). Treatment of the reaction product of the oxidative addition with py⋅HBF_4_ resulted in protonation of the ring‐nitrogen atom of the anionic CAAC ligand and formation of complex *trans*‐[**6**]BF_4_ bearing a unique protic CAAC ligand (*p*CAAC, Scheme 3, bottom).

**Scheme 3:**
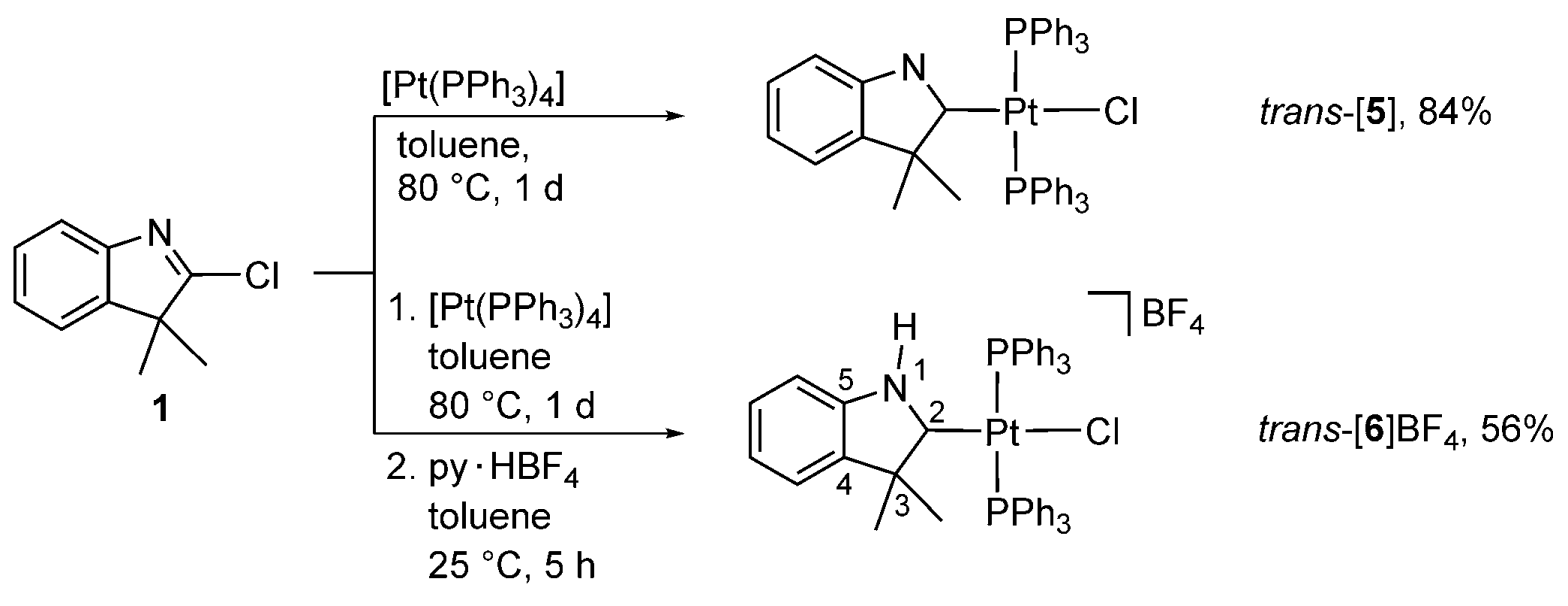
Synthesis of complexes *trans*‐[**5**] and *trans*‐[**6**]BF_4_.

The ^13^C NMR spectrum of *trans*‐[**5**] features the resonance for the carbene carbon atom as a triplet at *δ*(C2)=197.1 (t, ^2^ *J*_CP_=8.0 Hz), indicating the *trans*‐disposition of the phosphine donors. This resonance shifts significantly downfield to *δ*(C2)=221.4 (t, ^2^ *J*_CP_=7.6 Hz) in *trans*‐[**6**]BF_4_. In addition, the resonance for the N‐H proton was found in the ^1^H NMR spectrum of *trans*‐[**6**]BF_4_ at *δ*=12.33. Generally, all resonances in the NMR spectra shift downfield upon the transformation of *trans*‐[**5**] to *trans*‐[**6**]BF_4_ attributable to the introduction of a positive charge in *trans*‐[**6**]^+^. X‐Ray diffraction studies with crystals of *trans*‐[**5**]⋅CH_2_Cl_2_⋅C_6_H_14_ and *trans*‐[**6**]BF_4_⋅CH_2_Cl_2_ (Figure 3)^[16]^ confirmed the conclusions drawn from NMR spectroscopy.

**Figure 3:**
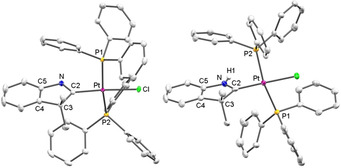
Molecular structures of complex *trans*‐[**5**] in *trans*‐[**5**]BF_4_⋅CH_2_Cl_2_⋅C_6_H_14_ (left) and of one of two essentially identical complex cations *trans*‐[**6**]^+^ in the asymmetric unit of *trans*‐[**6**]BF_4_⋅CH_2_Cl_2_ (right). Hydrogen atoms have been omitted for clarity except for H1 at N in *trans*‐[**6**]^+^. Thermal ellipsoids are set at 50 % probability.^[16]^ Selected bond lengths [Å] and angles (°) for *trans*‐[**5**] [*trans*‐[**6**]^+^]: Pt‐Cl 2.4056(5) [2.361(2)], Pt‐P1 2.3205(5) [2.3207(14)], Pt‐P2 2.3257(5) [2.332(2)], Pt‐C2 1.997(2) [1.974(8)], N‐C2 1.295(3) [1.302(10)], N‐C5 1.422(2) [1.428(10)], C2‐C3 1.561(3) [1.534(11)]; Cl‐Pt‐P1 87.17(2) [86.10(6)], Cl‐Pt‐P2 88.01(2) [87.01(7)], Cl‐Pt‐C2 174.09(6) [172.7(2)], P1‐Pt‐P2 164.76(2) [165.06(10)], P1‐Pt‐C2 93.50(6) [96.1(2)], P2‐Pt‐C2 92.77(6) [92.4(2)], C2‐N‐C5 108.1(2) [113.4(8)], N‐C2‐C3 113.1(2) [108.3(7)].

The protonation of the anionic CAAC ligand in *trans*‐[**5**] to give *trans*‐[**6**]^+^ has only a limited impact on comparable metric parameters. The most noticeable differences are the shrinkage of the N‐C2‐C3 angle from 113.1(2)° to 108.3(7)° and the expansion of the C2‐N‐C5 angle from 108.1(2)° to 113.4(8)° upon N‐protonation of *trans*‐[**5**]. Similar changes were observed upon protonation of coordinated azolato ligands to give complexes with *p*NHC ligands.^[15]^

Finally, 2‐chloroindole **1** was oxidatively added to [Pd(PPh_3_)_4_] in the presence of py⋅HBF_4_ to give *trans*‐[**7**]BF_4_ directly in a one‐pot reaction (Scheme 4). Compound *trans*‐[**7**]BF_4_ was fully characterized by NMR spectroscopy, ESI‐MS spectrometry and an X‐ray diffraction analysis (see the SI).^[16]^ The ^13^C NMR spectrum shows the resonance for the C_*p*CAAC_ carbon atom at *δ*(C2)=241.6 (t, ^2^ *J*_CP_=6.3 Hz) and the resonance for the N‐H proton was found at *δ*(H1)=12.45 in the ^1^H NMR spectrum. Comparable metric parameters in *trans*‐[**3**]BF_4_ (N‐ethyl substitution) and *trans*‐[**7**]BF_4_ (N‐H substitution) differ not significantly and confirm that the *p*CAAC in *trans*‐[**7**]^+^ behaves like a typical CAAC ligand.

**Scheme 4:**
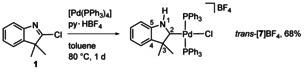
One**‐**pot synthesis of *p*CAAC complex *trans* **‐**[**7**]BF_4_.

We have prepared the 2‐chloroindole derived CAAC precursors **1** and **2**BF_4_. In contrast to the classical synthesis of CAAC complexes by deprotonation of aldiminium salts followed by CAAC coordination to a metal center, these precursors can oxidatively add to zero‐valent transition metals. This procedure gives access to complexes bearing CAAC ligand with N‐substituents previously unattainable. From precursor **1**, the first complexes bearing a CAAC ligand with an unsubstituted or a protonated ring‐nitrogen atom were obtained, while CAAC precursor **2**BF_4_ yielded complexes with N‐alkyl‐substituted CAAC ligands.

## Conflict of interest

The authors declare no conflict of interest.

## Supporting information

As a service to our authors and readers, this journal provides supporting information supplied by the authors. Such materials are peer reviewed and may be re‐organized for online delivery, but are not copy‐edited or typeset. Technical support issues arising from supporting information (other than missing files) should be addressed to the authors.

Supplementary
