# Supplementary material for: Preparation of Complexes Bearing N‐Alkylated, Anionic or Protic CAACs Through Oxidative Addition of 2‐Halogenoindole Derivatives
Source: Angew Chem Int Ed Engl. 2020 Nov 30;60(5):2599–602. doi: 10.1002/anie.202010988 (PMC7898591; doi:10.1002/anie.202010988)
Supplement: Supplementary file 1 — Supplementary [file ANIE-60-2599-s001.pdf]

Angewandte Chemie
Eine Zeitschrift der Gesellschaft Deutscher Chemiker

Supporting Information

Preparation of Complexes Bearing N-Alkylated, Anionic or Protic CAACs Through Oxidative Addition of 2-Halogenoindole Derivatives

*Sebastian Termühlen, Jonas Blumenberg, Alexander Hepp, Constantin G. Daniliuc, and F. Ekkehardt Hahn**

anie_202010988_sm_miscellaneous_information.pdf

Supporting Information

Contents

1. General procedures S2
2. Synthesis of CAAC precursors **1** and **2**BF_4 S2
3. Synthesis of complexes *cis*-[**3**]BF_4 and *trans*-[**3**]BF_4 S3
4. Synthesis of complex *cis*-[**4**]BF_4 S4
5. Synthesis of complex *trans*-[**5**] S4
6. Synthesis of complex *trans*-[**6**]BF_4 S5
7. Synthesis of complex *trans*-[**7**]BF_4 S5
8. X-ray crystallography S7
9. NMR spectra of all new compounds S11
10. References S21

1. General procedures

Unless stated otherwise, all manipulations were carried out under an argon atmosphere. ^{1}H and $^{13}C\{^{1}H\}$ and ^{31}P NMR spectra were measured on a Bruker AVANCE I 400, a Bruker AVANCE III 400 or a Bruker AVANCE Neo 500SB spectrometer. Chemical shifts (δ) are expressed in ppm relative to $SiMe_4$ using the residual protonated solvent signal as an internal standard. For the assignments of the NMR resonances see the numbering at the molecular plots. Coupling constants are expressed in Hz. Mass spectra were obtained with an Orbitrap LTQ XL spectrometer (Thermo Scientific). Compounds 2-chloro-3,3-dimethylindole[S1] and 2-chloro-1-ethyl-3,3-dimethylindolium tetrafluoroborate[S2] and were prepared following published procedures.

2. Synthesis of CAAC precursors 1 and 2BF$_4$

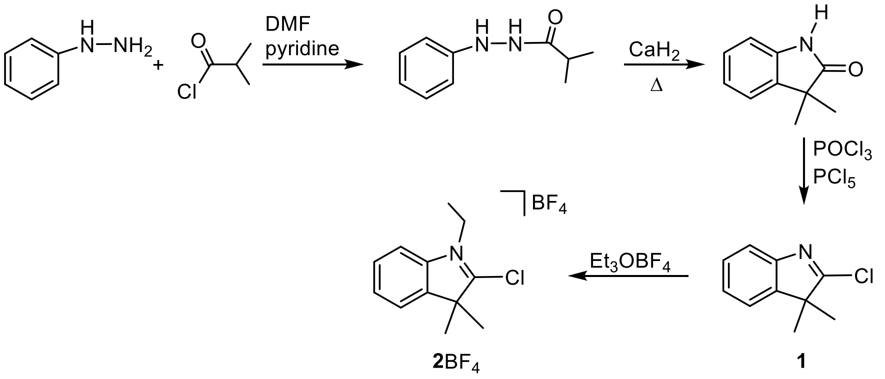

Scheme S1. Synthesis of 1 and 2BF$_4$

2.1. Synthesis of 1

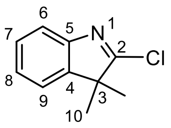

Compound **1** was prepared by reaction of phenylhydrazine with isobutyryl chloride followed by cyclization with CaH$_2$.[S1] The 3,3-dimethylindolinon was subsequently converted into the 2-chloro-3,3-dimethylindole **1** by reaction with POCl$_3$/PCl$_5$.[S2] ^{1}H NMR (400 MHz, CDCl$_3$): δ (ppm) = 7.51 (m, 1H, Ar-H), 7.33–7.17 (m, 3H, Ar-H), 1.33 (s, 6H, CH$_3$).

2.2. Synthesis of 2BF$_4$

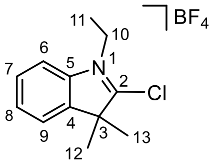

N-Alkylation of 2-chloro-3,3-dimethylindole **1** with Et$_3$OBF$_4$ yielded 2-chloro-1-ethyl-3,3-dimethylindolium tetrafluoroborate as hygroscopic colorless solid.[S2] ^{1}H NMR (400 MHz, CD$_3$CN): δ (ppm) = 7.87–7.83 (m, 1H, Ar-H), 7.80–7.65 (m, 3H, Ar-H), 4.56 (t, $^3J_{HH}$ = 7.4 Hz, 2H, CH$_2$), 1.64 (s, 6H, C–CH$_3$), 1.56 (t, $^3J_{HH}$ = 7.4 Hz, 3H, CH$_2$–CH_3).

3. Synthesis of complexes *cis*-[3]BF$_4$ and *trans*-[3]BF$_4$

3.1. Synthesis of *cis*-[3]BF$_4$

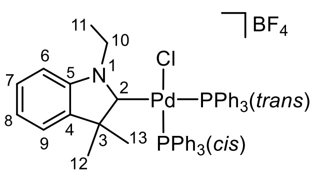

A suspension of 2-chloro-1-ethyl-3,3-dimethylindolium tetrafluoroborat **2**BF$_4$ (15 mg, 0.05 mmol) and [Pd(PPh$_3$)$_4$] (58 mg, 0.05 mmol) in toluene (5 mL) was stirred at 25 °C for 4 d. Subsequently, the suspension was filtered and the isolated solid was washed with toluene (3 × 5 mL), diethyl ether (3 × 5 mL) and ice-cold aceton (1 mL). The solid was dried *in vacuo* to give *cis*-[**3**]BF$_4$ as a colorless solid. Yield: 40.6 mg (0.044 mmol, 88%). ^{1}H NMR (400 MHz, CD$_2$Cl$_2$): δ (ppm) = 7.71–7.09 (multiple m, 34 H, Ph-H$_{ortho,cis}$, Ph-H$_{para,cis}$, Ph-H$_{para,trans}$, H7, Ph-H$_{ortho,trans}$, H8, Ph-H$_{meta,cis}$, H9, Ph-H$_{meta,trans}$, H6), 5.16 (dq, $^2J_{HH}$ = 13.4, $^3J_{HH}$ = 7.2 Hz, 1H, H10), 4.26 (dq, $^2J_{HH}$ = 13.4, $^3J_{HH}$ = 7.2 Hz, 1H, H10‘), 1.86 (t, $^3J_{HH}$ = 7.2 Hz, 3H, H11), 1.72 (s, 3H, H12), 1.07 (s, 3H, H13). ^{13}C{^{1}H} NMR (101 MHz, CD$_2$Cl$_2$): δ (ppm) = 242.2 (dd, $^2J_{CP,trans}$ = 141.9, $^2J_{CP,cis}$ = 5.7 Hz, C2), 146.8 (d, $^4J_{CP,trans}$

= 4.2 Hz, C4), 142.6 (d, $^4J_{CP,trans}$ = 8.2 Hz, C5), 135.2 (d, $^2J_{CP}$ = 10.4 Hz, Ph-$C_{ortho,trans}$), 135.0 (br, Ph-$C_{ortho,cis}$), 133.1 (br, Ph-$C_{para,cis}$), 132.3 (br, Ph-$C_{ipso,cis}$), 131.4 (d, $^4J_{CP}$ = 2.5 Hz, Ph-$C_{para,trans}$), 129.8 (d, $^1J_{CP}$ = 45.0 Hz, Ph-$C_{ipso,trans}$), 129.7 (br, Ph-$C_{meta,cis}$), 128.9 (d, $^3J_{CP}$ = 10.3 Hz, Ph-$C_{meta,trans}$), 128.3 (C7), 127.7 (C8), 122.6 (C9), 113.4 (C6), 61.9 (dd, $^3J_{CP,trans}$ = 6.9, $^3J_{CP,cis}$ = 1.2 Hz, C3), 51.8 (C10), 30.1 (C12), 24.4 (d, $^4J_{CP,trans}$ = 1.4 Hz, C13), 12.9 (C11). $^{31}P\{^1H\}$ NMR (162 MHz, CD_2Cl_2): δ(ppm) = 29.8 (d, $^2J_{PP}$ = 26.0 Hz, P_{trans}), 19.6 (d, $^2J_{PP}$ = 26.0 Hz, P_{cis}). HRMS (ESI, positive ions): *m/z* (%) = 838.1754 (100, calcd for [**3**]$^+$ = 838.1765).

3.2. Synthesis of *trans*-[3]BF4

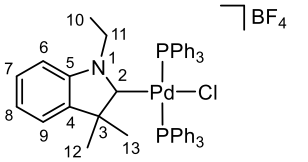

Complex *trans*-[**3**]BF_4 was obtained as the main product (about 60%) together with *cis*-[**3**]BF_4 and some decomposition products such as [PdCl(PPh_3)$_3$] when **2**BF_4 (15 mg, 0.05 mmol) and [Pd(PPh_3)$_4$] were reacted as described for the synthesis of *cis*-[**3**]BF_4 but using a reaction temperature of 80 °C (instead of 25 °C) for 1 d. The reaction products could not be separated and thus no detailed NMR spectroscopic characterization was possible. The $^{31}P\{^1H\}$ NMR spectrum (162 MHz, CD_2Cl_2) shows the formation of three reaction products at δ(ppm) = 32.2 (t, $^2J_{PP}$ = 15.3 Hz, P *trans* to Cl in [PdCl(PPh_3)$_3$]BF_4), 29.8 (d, $^2J_{PP}$ = 26.0 Hz, P_{trans} in *cis*-[**3**]BF_4), 29.5 (d, $^2J_{PP}$ = 15.3 Hz, P *cis* to Cl in [PdCl(PPh_3)$_3$]BF_4), 24.2 (s, P in *trans*-[**3**]BF_4), 19.6 (d, $^2J_{PP}$ = 26.0 Hz, P_{cis} in *cis*-[**3**]BF_4). Crystallization of the complex mixture *trans*-[**3**]BF_4, *cis*-[**3**]BF_4 and [PdCl(PPh_3)$_3$]BF_4 from CH_2Cl_2 yielded yellow crystals of slightly different shapes allowing the isolation of a crystal of *trans*-[**3**]BF_4 for a subsequent X-ray structure determination.

4. Synthesis of complex *cis*-[4]BF4

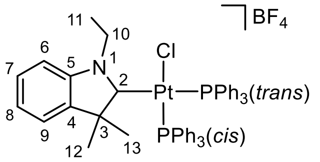

A suspension of 2-chloro-1-ethyl-3,3-dimethylindolium tetrafluoroborat **2**BF_4 (15 mg, 0.05 mmol) and [Pt(PPh_3)$_4$] (62 mg, 0.05 mmol) in toluene (5 mL) was stirred at 110 °C for 1 h. The suspension was subsequently filtered and the isolated solid was washed with toluene (3 × 5 mL) and diethyl ether (3 × 5 mL). The solid was dried *in vacuo* to give compound *cis*-[**4**]BF_4 as a colorless solid. Yield: 35.8 mg (0.035 mmol, 70%). ^{1}H NMR (400 MHz, CD_2Cl_2): δ(ppm) = 7.78–6.95 (multiple m, 34 H, Ph-$H_{ortho,cis}$, Ph-$H_{para,cis}$, Ph-$H_{para,trans}$, H7, Ph-$H_{ortho,trans}$, Ph-$H_{meta,cis}$, H8, H6, H9, Ph-$H_{meta,trans}$), 5.37–5.32 (partly overlapped m, 1H, H10), 4.18 (dq, $^2J_{HH}$ = 14.2, $^3J_{HH}$ = 7.2 Hz, 1H, H10‘), 1.85 (t, $^3J_{HH}$ = 7.2 Hz, 3H, H11), 1.69 (s, 3H, H12), 0.99 (s, 3H, H13). $^{13}C\{^1H\}$ NMR (101 MHz, CD_2Cl_2): δ(ppm) = 232.9 (dd, $^2J_{CP,trans}$ = 126.7, $^2J_{CP,cis}$ = 8.2 Hz, C2), 146.3 (d, $^4J_{CP,trans}$ = 3.5 Hz, C4), 142.9 (d, $^4J_{CP,trans}$ = 7.1 Hz, C5), 135.4 (br, Ph-$C_{ortho,trans}$), 135.1 (br, Ph-$C_{ortho,cis}$), 133.1 (br, Ph-$C_{para,cis}$), 132.1 (br, Ph-

$C_{ipso,cis}$), 131.5 (d, $^{4}J_{CP}$ = 2.5 Hz, Ph-$C_{para,trans}$), 129.5 (br, Ph-$C_{meta,cis}$), 129.3 (d, $^{1}J_{CP}$ = 54.6 Hz, Ph-$C_{ipso,trans}$), 128.8 (d, $^{3}J_{CP}$ = 10.6 Hz, Ph-$C_{meta,trans}$), 128.4 (C7), 127.8 (C8), 122.8 (C9), 113.5 (C6), 61.9 (d, $^{3}J_{CPtrans}$ = 6.3 Hz, C3), 50.8 (C10), 29.5 (C12), 24.4 (C13), 12.9 (C11). $^{31}P\{^{1}H\}$ NMR (162 MHz, CD_2Cl_2): δ (ppm) = 15.5 (d, $^{2}J_{PP}$ = 20.4 Hz; d, $^{1}J_{PPt}$ = 2020 Hz, Pt satellites, P_{trans}), 11.1 (d, $^{2}J_{PP}$ = 20.4 Hz; d, $^{1}J_{PPt}$ = 3773 Hz, Pt satellites, P_{cis}). ^{195}Pt NMR (107 MHz, CD_2Cl_2): δ (ppm) = −4248 (dd, $^{1}J_{PtPcis}$ = 3773, $^{1}J_{PtPtrans}$ = 2020 Hz). HRMS (ESI, positive ions): *m/z* (%) = 928.2363 (100, calcd for $[\mathbf{4}]^+$ = 928.2369).

5. Synthesis of complex *trans*-[5]

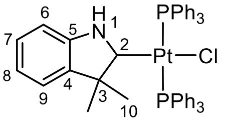

A solution of 2-chloro-3,3-dimethylindole **1** (9 mg, 0.05 mmol) and $[Pt(PPh_3)_4]$ (62 mg, 0.05 mmol) in toluene (5 mL) was stirred at 80 °C for 1d. The solution was then left at –30 °C for 40 h leading to precipitation of a colorless solid. The solid was isolated by filtration. It was washed with diethyl ether (3 × 5 mL) and dried *in vacuo* to give *trans*-[**5**] as a colorless solid. Yield: 37.4 mg (0.042 mmol, 84%). ^{1}H NMR (400 MHz, CD_2Cl_2): δ (ppm) = 7.78–7.71 (m, 12H, Ph-H_{ortho}), 7.41–7.34 (m, 6H, Ph-H_{para}), 7.33–7.25 (m, 13H, Ph_{meta} and H6), 7.05 (td, $^{3}J_{HH}$ = 7.5 Hz, $^{4}J_{HH}$ = 1.4 Hz, 1H, H7), 6.73 (td, $^{3}J_{HH}$ = 7.3 Hz, $^{4}J_{HH}$ = 0.9 Hz, 1H, H8), 6.67 (dd, $^{3}J_{HH}$ = 7.3 Hz, $^{4}J_{HH}$ = 1.4 Hz, 1H, H9), 0.44 (s, 6H, H10). $^{13}C\{^{1}H\}$ NMR (101 MHz, CD_2Cl_2): δ (ppm) = 197.1 (t, $^{2}J_{CP}$ = 8.0 Hz, C2), 158.2 (C5), 148.3 (C4), 135.8 (v-t, $^{2/4}J_{CP}$ = 6.0 Hz, Ph-C_{ortho}), 131.0 (Ph-C_{para}), 131.0 (v-t, $^{1/3}J_{CP}$ = 28.2 Hz, Ph-C_{ipso}), 128.4 (v-t, $^{3/5}J_{CP}$ = 5.3 Hz, Ph-V_{meta}), 126.5 (C7), 122.1 (C8), 120.3 (C9), 117.6 (C6), 62.20 (t, $^{3}J_{CP}$ = 1.5 Hz, C3), 25.45 (C10). $^{31}P\{^{1}H\}$ NMR (162 MHz, CD_2Cl_2): δ (ppm) = 18.6 (s; d, $^{1}J_{PPt}$ = 3146 Hz, Pt satellites). ^{195}Pt NMR (107 MHz, CD_2Cl_2): δ (ppm) = −4237 (t, $^{1}J_{PtP}$ = 3146 Hz). HRMS (ESI, positive ions): *m/z* (%) = 900.2044 (100, calcd for $[\mathbf{5}+H]^+$ = 900.2056).

6. Synthesis of complex *trans*-[6]BF_4

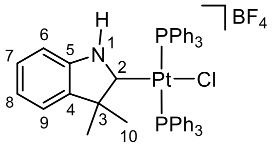

A solution of 2-chloro-3,3-dimethylindole **1** (9 mg, 0.05 mmol) and $[Pt(PPh_3)_4]$ (62 mg, 0.05 mmol) in toluene (5 mL) was stirred at 80 °C for 1d. Subsequently, py·HBF_4 (9 mg, 0.05 mmol) was added and the suspension was stirred at 25 °C for 5 h. The suspension was then filtered and the isolated solid was washed with toluene (3 × 5 mL) and diethyl ether (3 × 5 mL). The solid was then dried *in vacuo* to give *trans*-[**6**]BF_4 as a colorless solid. Yield: 28.0 mg (0.028 mmol, 56%). ^{1}H NMR (400 MHz, CD_2Cl_2): δ (ppm) = 12.33 (s, 1H, H1), 7.92–7.83 (m, 12H, Ph-H_{ortho}), 7.47–7.39 (m, 18H, Ph-H_{meta} and Ph-H_{para}), 7.26 (d, $^{3}J_{HH}$ = 7.3 Hz, 1H, H6), 7.13 (td, $^{3}J_{HH}$ = 7.7, $^{4}J_{HH}$ = 1.2 Hz, 1H, H7), 7.00 (td, $^{3}J_{HH}$ =

7.5, ${}^{4}J_{HH}$ = 1.0 Hz, 1H, H8), 6.87 (d, ${}^{3}J_{HH}$ = 7.4 Hz, 1H, H9), 0.85 (s, 6H, H10). ${}^{13}C\{{}^{1}H\}$ NMR (101 MHz, CD_2Cl_2): δ (ppm) = 221.4 (t, ${}^{2}J_{CP}$ = 7.6 Hz, C2), 143.5 (C4), 142.9 (C5), 135.4 (v-t, ${}^{2/4}J_{CP}$ = 6.1 Hz, Ph-C*ortho*), 131.9 (Ph-C*para*), 129.0 (v-t, ${}^{3/5}J_{CP}$ = 5.5 Hz, Ph-C*meta*), 128.6 (v-t, ${}^{1/3}J_{CP}$ = 20.4 Hz, Ph-C*ipso*), 127.7 (C7), 126.1 (C8), 121.5 (C9), 114.9 (C6), 62.8 (C3), 26.4 (C10). ${}^{31}P\{{}^{1}H\}$ NMR (162 MHz, CD_2Cl_2): δ (ppm) = 18.8 (s; d, ${}^{1}J_{PPt}$ = 2674 Hz, Pt satellites). ${}^{195}Pt$ NMR (107 MHz, CD_2Cl_2): δ (ppm) = −4192 (t, ${}^{1}J_{PPt}$ = 2674 Hz). HRMS (ESI, positive ions): *m*/*z* (%) = 900.2057 (100, calcd for [**6**]$^{+}$ = 900.2056).

7. Synthesis of complex *trans*-[7]BF_4

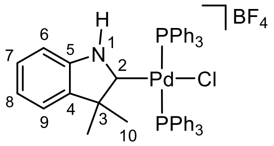

A suspension of 2-chloro-3,3-dimethylindole **1** (9 mg, 0.05 mmol), pyr·HBF_4 (9 mg, 0.05 mmol) and [Pd(PPh_3)$_4$] (58 mg, 0.05 mmol) in toluene (5 mL) was stirred at 80 °C for 1d. The resulting suspension was filtered and the isolated solid was washed with toluene (3 × 5 mL) and diethyl ether (3 × 5 mL) and dried *in vacuo* to give *trans*-[**7**]BF_4 as a colorless solid. Yield: 30.7 mg (0.034 mmol, 68%). ${}^{1}H$ NMR (400 MHz, CD_2Cl_2): δ (ppm) = 12.45 (s, 1H, H1), 7.91–7.76 (m, 12H, Ph-H*ortho*), 7.50–7.35 (m, 18H, Ph-H*meta* and Ph-H*para*), 7.27 (d, ${}^{3}J_{HH}$ = 7.9 Hz, 1H, H6), 7.13 (td, ${}^{3}J_{HH}$ = 7.7, ${}^{4}J_{HH}$ = 1.2 Hz, 1H, H7), 7.03 (td, ${}^{3}J_{HH}$ = 7.5, ${}^{4}J_{HH}$ = 1.0 Hz, 1H, H8), 6.92 (d, ${}^{3}J_{HH}$ = 7.3 Hz, 1H, H9), 0.95 (s, 6H, H10). ${}^{13}C\{{}^{1}H\}$ NMR (101 MHz, CD_2Cl_2): δ (ppm) = 241.6 (t, ${}^{2}J_{CP}$ = 6.3 Hz, C2), 144.3 (C4), 142.7 (C5), 135.2 (v-t, ${}^{2/4}J_{CP}$ = 6.1 Hz, Ph-C*ortho*), 131.7 (Ph-C*para*), 129.4 (v-t, ${}^{1/3}J_{CP}$ = 25.2 Hz, Ph-C*ipso*), 129.1 (v-t, ${}^{3/5}J_{CP}$ = 5.3 Hz, Ph-C*meta*), 127.7 (C7), 126.4 (C8), 121.2 (C9), 115.1 (C6), 62.7 (t, ${}^{3}J_{CP}$ = 1.4 Hz, C3), 26.0 (t, ${}^{4}J_{CP}$ = 1.7 Hz, C10). ${}^{31}P\{{}^{1}H\}$ NMR (162 MHz, CD_2Cl_2): δ (ppm) = 22.5 (s). HRMS (ESI, positive ions): *m*/*z* (%) = 810.1445 (73, calcd for [**7**]$^{+}$ = 810.1451).

8. X-ray crystallography

X-ray diffraction data were collected with a Bruker AXS APEXII CCD diffractometer (*cis*-[**4**]BF_4 and *trans*-[**7**]BF_4) or with a Bruker D8 Venture PHOTON 100 diffractometer (and *cis*-[**3**]BF_4, *trans*-[**3**]BF_4, *trans*-[**5**] and *trans*-[**6**]BF_4). Semiempirical multi-scan absorption corrections were applied to all data sets.[S3,S4] Structure solutions were found with SHELXT (intrinsic phasing)[S5] and were refined with SHELXL[S6] against $|F^2|$ of all data using first isotropic and later anisotropic thermal parameters for all non-hydrogen atoms. Hydrogen atoms have been added to the structure models on calculated positions.

Exceptions and special features: For compound *trans*-[**5**] one dichloromethane molecule and for compound *trans*-[**6**]BF_4 one BF_4 anion were found disordered over two positions in the asymmetric unit. Several restraints (SADI, SAME, ISOR and SIMU) were used in order to improve refinement stability. For compounds *cis*-[**3**]BF_4 and *cis*-[**4**]BF_4 two badly disordered dichloromethane molecules

were found in the asymmetrical unit and could not be satisfactorily refined. The program SQUEEZE was used to remove mathematically the effect of the solvent molecules. The quoted formula and derived parameters are not included the squeezed solvent molecules. Compounds *trans*-[**6**]BF_4 and *trans*-[**7**]BF_4 crystallized in the chiral space group $P2_1$ of the monoclinic crystal system with two independent molecules in the asymmetric unit. Tests for a possible higher (pseudo) symmetry using the PLATON/ADDSYM were done.

*Crystal data for cis-[**3**]BF_4.* Single crystals were grown at ambient temperature form a CH_2Cl_2 solution of *cis*-[**3**]BF_4 layered with hexane. Crystal data for *cis*-[**3**]BF_4: formula $C_{48}H_{45}NBClF_4P_2Pd$, M = 926.45 g·mol^{-1}, colorless block, 0.22 × 0.19 × 0.08 mm^3, a = 13.0776(5), b = 14.1653(5), c = 26.8858(11) Å, β = 101.236(2)°, V = 4885.10(3) Å^3, Mo Kα radiation, ρ_{calc} = 1.260 g·cm^{-3}, μ = 0.546 mm^{-1}, monoclinic, space group $P2_1/c$, Z = 4, empirical absorption correction (0.90 ≤ T ≤ 0.96), T = 100(2) K, ω and φ scans, 65673 intensities collected (4.3° ≤ 2θ ≤ 55.0°), 11196 unique intensities (R_{int} = 0.054) and 10103 observed intensities ($I \geq 2\sigma(I)$), refinement of 526 parameters against $|F^2|$ of all independent intensities with hydrogen atoms on calculated positions. R = 0.0434, R_w = 0.0873, R_{all} = 0.0493, $R_{w,all}$ = 0.0898. The asymmetric unit contains one formula unit *cis*-[**3**]BF_4 and two badly disordered dichloromethane molecules squeezed (see above *Exceptions and special features*).

*Crystal data for trans-[**3**]BF_4·CH_2Cl_2.* Single crystals were grown at ambient temperature by diffusion of diethyl ether into a CH_2Cl_2 solution of a complex mixture of *trans*-[**3**]BF_4, *cis*-[**3**]BF_4 and [PdCl(PPh_3)$_3$] in CH_2Cl_2. Crystal data for *trans*-[**3**]BF_4·CH_2Cl_2: formula $C_{49}H_{47}NBCl_3F_4P_2Pd$, M = 1011.37 g·mol^{-1}, yellow plate, 0.16 × 0.15 × 0.08 mm^3, a = 15.2806(3), b = 15.9434(4), c = 18.3494(4) Å, V = 4470.4(2) Å^3, Mo Kα radiation, ρ_{calc} = 1.503 g·cm^{-3}, μ = 0.719 mm^{-1}, orthorhombic, space group $P2_12_12_1$, Z = 4, empirical absorption correction (0.89 ≤ T ≤ 0.94), T = 100(2) K, ω and φ scans, 36346 intensities collected (5.1° ≤ 2θ ≤ 53.5°), 9501 unique intensities (R_{int} = 0.052) and 8943 observed intensities ($I \geq 2\sigma(I)$), refinement of 553 parameters against $|F^2|$ of all independent intensities with hydrogen atoms on calculated positions. R = 0.0304, R_w = 0.0617, R_{all} = 0.0341, $R_{w,all}$ = 0.0634. The asymmetric unit contains one formula unit *trans*-[**3**]BF_4 and one molecule of CH_2Cl_2.

*Crystal data for cis-[**4**]BF_4.* Single crystals were grown at ambient temperature from a CH_2Cl_2 solution of *cis*-[**4**]BF_4. Crystal data for *cis*-[**4**]BF_4: formula $C_{48}H_{45}NBClF_4P_2Pt$, M = 1015.14 g·mol^{-1}, colorless block, 0.16 × 0.08 × 0.02 mm^3, a = 13.0205(2), b = 14.2077(3), c = 26.2101(5) Å, β = 99.944(1)°, V = 4775.8(2) Å^3, Cu Kα radiation, ρ_{calc} = 1.412 g·cm^{-3}, μ = 7.031 mm^{-1}, monoclinic, space group $P2_1/c$, Z = 4, empirical absorption correction (0.67 ≤ T ≤ 0.87), T = 100(2) K, ω and φ

scans, 54386 intensities collected (7.1° ≤ 2θ≤ 134.0°), 8432 unique intensities (R_{int} = 0.102) and 6676 observed intensities ($I \geq 2\sigma(I)$), refinement of 526 parameters against $|F^2|$ of all independent intensities with hydrogen atoms on calculated positions. R = 0.0365, R_w = 0.0814, R_{all} = 0.0509, $R_{w,all}$ = 0.0860. The asymmetric unit contains one formula unit of *cis*-[**4**]BF_4 and two badly disordered dichloromethane molecules squeezed (see above *Exceptions and special features*).

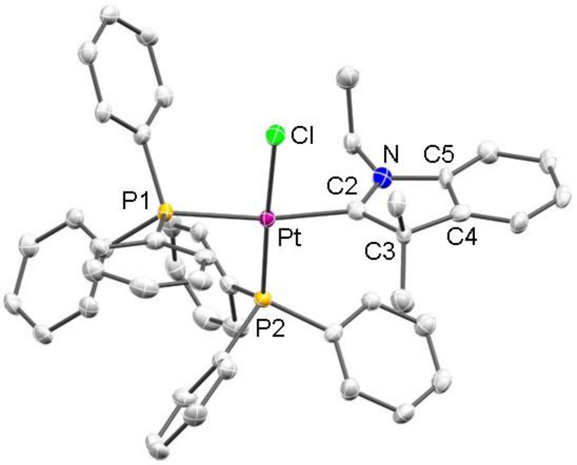

Figure S1. Molecular structures of complex cation *cis*-[**4**]$^+$ in *cis*-[**4**]BF_4. Hydrogen atoms have been omitted for clarity and 50% probability ellipsoids are depicted. Selected bond lengths (Å) and angles (deg): Pt–Cl 2.544(11), Pt–P1 2.3684(12), Pt–P2 2.2657(12), Pt–C2 2.029(5), N–C2 1.304(6), C2–C3 1.527(7); Cl–Pt–P1 83.62(4), Cl–Pt–P2 177.88(4), Cl–Pt–C2 84.19(13), P1–Pd–P2 98.50(4), P1–Pt–C2 167.65(13), P2–Pt–C2 93.69(13), C2–N–C5 112.5(4), N–C2–C3 108.8(4).

*Crystal data for trans-[**5**]·CH_2Cl_2·C_6H_{14}*. Single crystals were grown at ambient temperature by layering a CH_2Cl_2 solution of *trans*-[**5**]·CH_2Cl_2·C_6H_{14} with hexane. Crystal data for *trans*-[**5**]·CH_2Cl_2·C_6H_{14}: formula $C_{53}H_{56}NCl_3P_2P1$, M = 1070.36 g·mol^{-1}, colorless block, 0.24 × 0.16 × 0.10 mm^3, a = 10.3561(2), b = 13.4069(3), c = 17.7234(3) Å, α = 76.000(1), β = 88.745(1)°, γ = 83.643(1)°, V = 2372.97(8) Å^3, Mo Kα radiation, ρ_{calc} = 1.498 g·cm^{-3}, μ = 3.230 mm^{-1}, triclinic, space group P–1, Z = 2, empirical absorption correction (0.70 ≤ T ≤ 0.78), T = 100(2) K, ω and φ scans, 49756 intensities collected (4.6° ≤ 2θ ≤ 55.0°), 10846 unique intensities (R_{int} = 0.040) and 10176 observed intensities ($I \geq 2\sigma(I)$), refinement of 564 parameters against $|F^2|$ of all independent intensities with hydrogen atoms on calculated positions. R = 0.0192, R_w = 0.0403, R_{all} = 0.0219, $R_{w,all}$ = 0.0412. The asymmetric unit contains one formula unit *trans*-[**5**], one molecule of CH_2Cl_2 and one molecule of C_6H_{14}.

*Crystal data for trans-[**6**]BF_4·CH_2Cl_2*. Single crystals of *trans-[**6**]BF_4·CH_2Cl_2* were grown at ambient

temperature layering a CH_2Cl_2 solution of *trans*-[**6**]BF_4 with hexane. Crystal data for *trans*-[**6**]$BF_4 \cdot CH_2Cl_2$: formula $C_{47}H_{43}NBCl_3F_4P_2Pt$, M = 1072.01 g·mol^{-1}, colorless plate, 0.37 × 0.21 × 0.06 mm^3, a = 20.4652(14), b = 10.8329(8), c = 20.7960(16) Å, β = 106.561(3)°, V = 4419.20(6) Å^3, Mo Kα radiation, ρ_{calc} = 1.611 g·cm^{-3}, μ = 3.481 mm^{-1}, monoclinic, space group $P2_1$, Z = 4, empirical absorption correction ($0.60 \leq T \leq 0.82$), T = 100(2) K, ω and φ scans, 122159 intensities collected ($4.5° \leq 2\theta \leq 55.0°$), 20166 unique intensities (R_{int} = 0.070) and 16881 observed intensities ($I \geq 2\sigma(I)$), refinement of 1116 parameters against $|F^2|$ of all independent intensities with hydrogen atoms on calculated positions. R = 0.0329, R_w = 0.0640, R_{all} = 0.0507, $R_{w,all}$ = 0.0744. The asymmetric unit contains two essentially identical formula units of *trans*-[**6**]BF_4 and two molecules of CH_2Cl_2.

*Crystal data for trans-[**7**]$BF_4 \cdot CH_2Cl_2$.* Single crystals of *trans-[**7**]BF_4* were grown at ambient temperature layering a CH_2Cl_2 solution of *trans*-[**7**]BF_4 with hexane. Crystal data for *trans*-[**7**]$BF_4 \cdot CH_2Cl_2$: formula $C_{47}H_{43}NBCl_3F_4P_2Pd$, M = 983.32 g·mol^{-1}, colorless block, 0.20 × 0.12 × 0.12 mm^3, a = 20.5739(8), b = 10.7911(4), c = 20.7923(7) Å, β = 107.133(2)°, V = 4411.40(3) Å^3, Cu Kα radiation, ρ_{calc} = 1.481 g·cm^{-3}, μ = 6.179 mm^{-1}, monoclinic, space group $P2_1$, Z = 4, empirical absorption correction ($0.38 \leq T \leq 0.52$), T = 100(2) K, ω and φ scans, 79496 intensities collected ($4.4° \leq 2\theta \leq 133.7°$), 15536 unique intensities (R_{int} = 0.126) and 12194 observed intensities ($I \geq 2\sigma(I)$), refinement of 1071 parameters against $|F^2|$ of all independent intensities with hydrogen atoms on calculated positions. R = 0.0510, R_w = 0.1182, R_{all} = 0.0726, $R_{w,all}$ = 0.1317. The asymmetric unit contains two essentially identical formula units of *trans*-[**7**]BF_4 and two molecules of CH_2Cl_2.

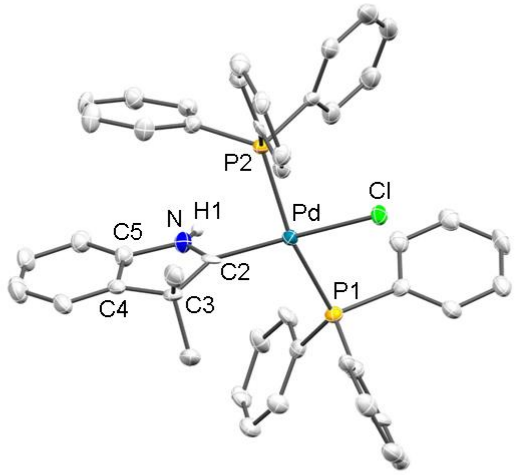

Figure S2. Molecular structures of one of the two essentially identical complex cations *trans*-[**7**]$^+$ in the asymmetric unit of *trans*-[**7**]$BF_4 \cdot CH_2Cl_2$. Hydrogen atoms have been omitted for clarity and 50% probability ellipsoids are depicted. Selected bond lengths (Å) and angles (deg): Pd–Cl 2.356(2), Pd–P1 2.338(2), Pd–P2 2.352(2), Pd–C2 1.989(10), N–C2 1.286(13), N–C5 1.434(13), C2–C3 1.533(14); Cl–Pd–P1 87.28(8), Cl–Pd–P2 87.00(8), Cl–Pd–C2 171.3(3), P1–Pd–P2 164.47(10), P1–Pd–C2

96.0(3), P2–Pd–C2 91.8(3), C2–N–C5 113.6(10), N–C2–C3 108.9(9).

9. NMR spectra of all new compounds

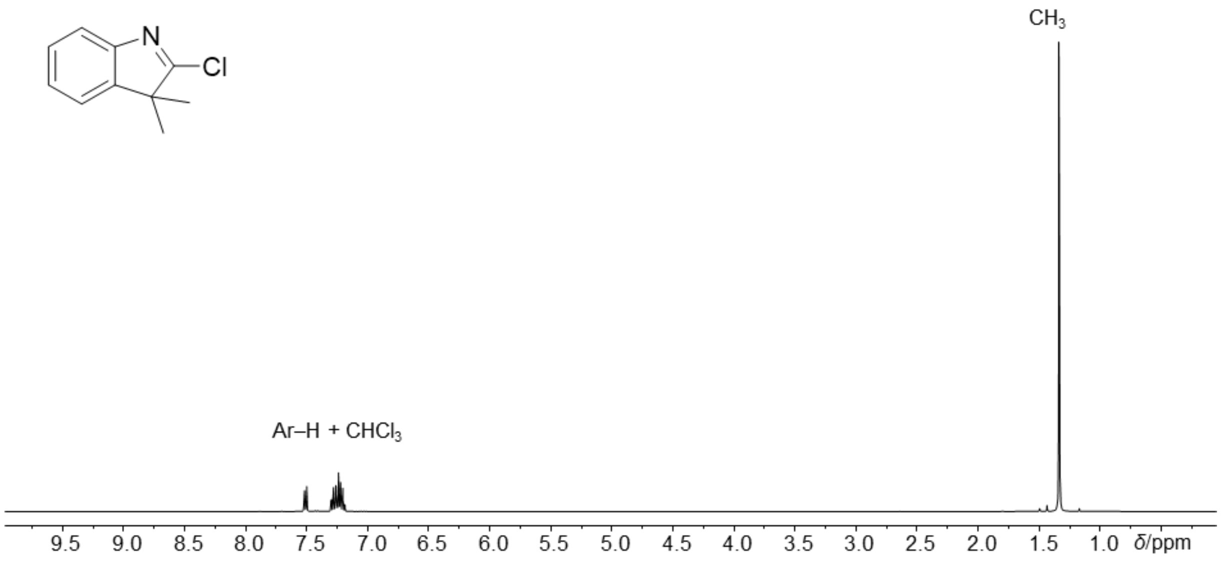

Figure S3. ^{1}H NMR spectrum of 2-chloro-3,3-dimethylindole **1** (400 MHz, $CDCl_3$).

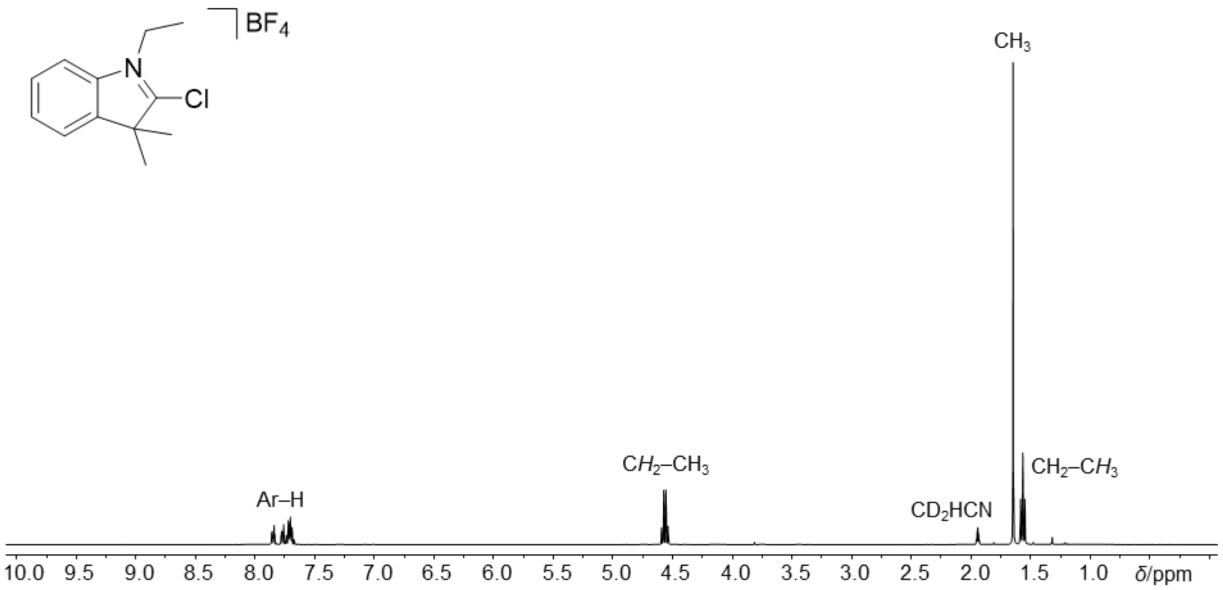

Figure S4. ^{1}H NMR spectrum of 2-chloro-1-ethyl-3,3-dimethylindolium tetrafluoroborate **2**[BF_4] (400 MHz, CD_3CN).

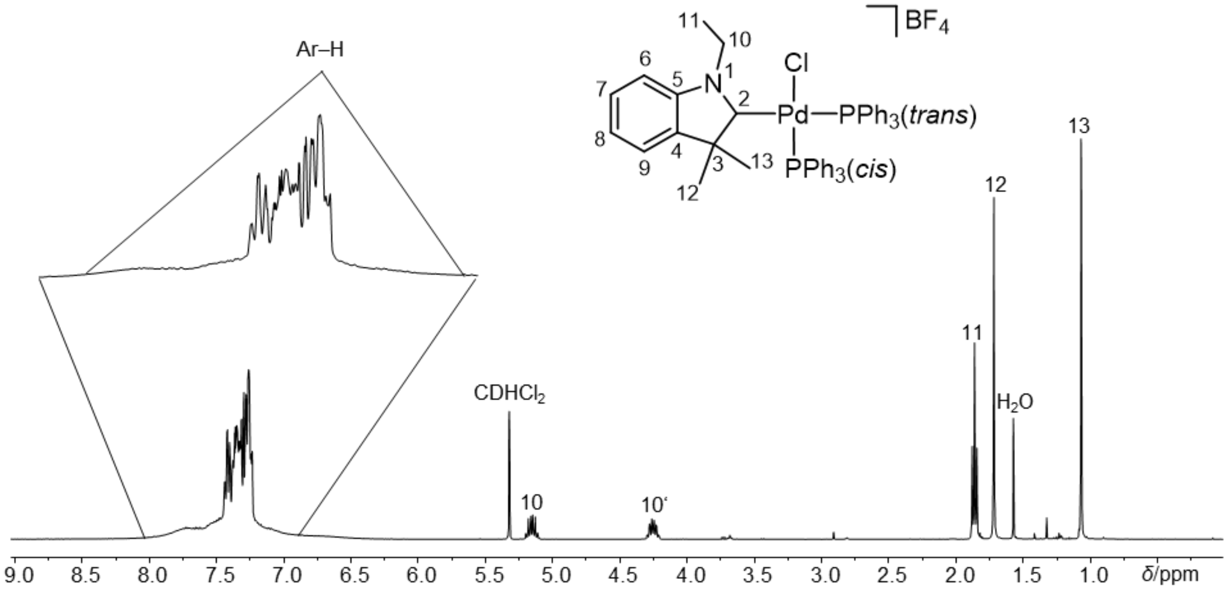

Figure S5. ^{1}H NMR spectrum of *cis*-[**3**]BF_4 (400 MHz, CD_2Cl_2).

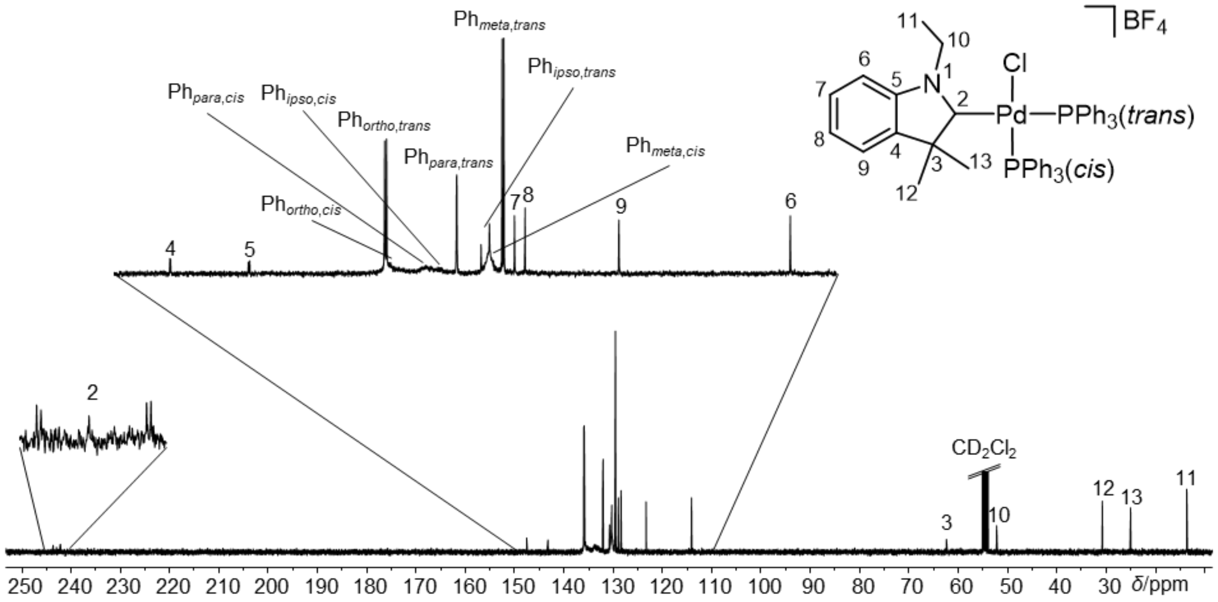

Figure S6. ^{13}C NMR spectrum of *cis*-[**3**]BF_4 (101 MHz, CD_2Cl_2).

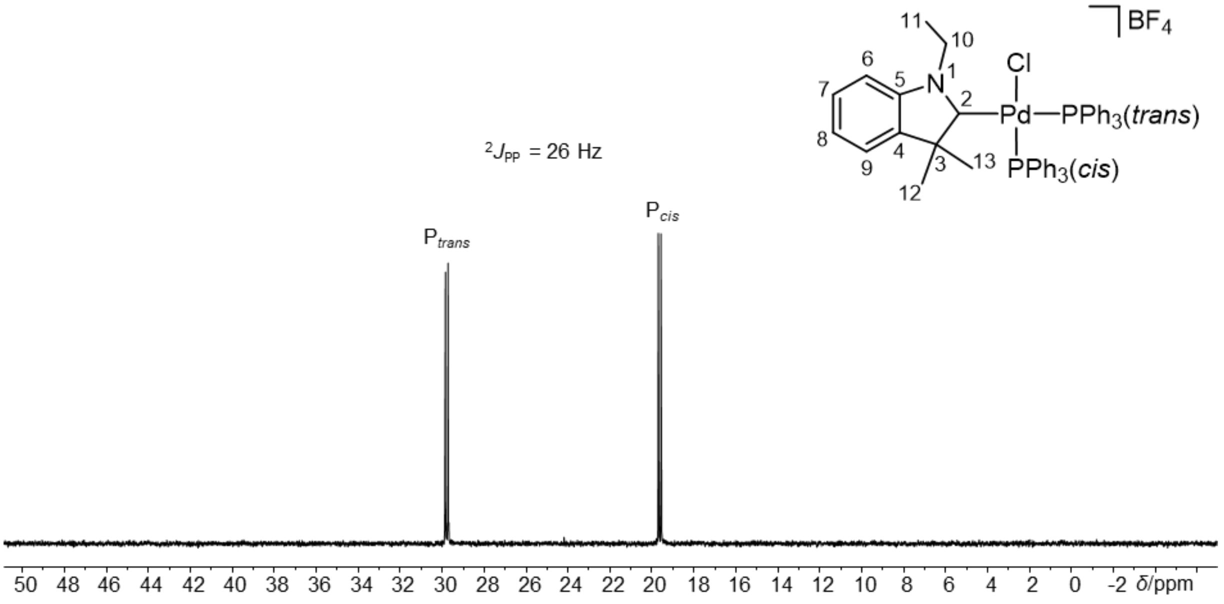

Figure S7. $^{31}P\{^1H\}$ NMR spectrum of *cis*-[**3**]BF_4 (162 MHz, CD_2Cl_2).

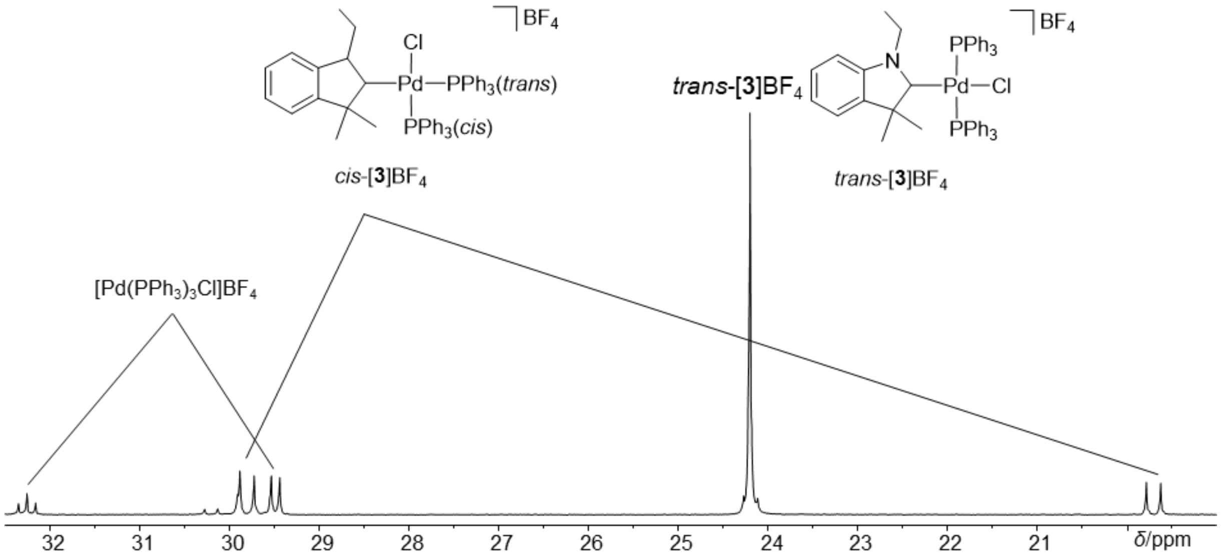

Figure S8. $^{31}P\{^{1}H\}$ NMR spectrum of the complex mixture *cis*-[**3**]BF_4, *trans*-[**3**]BF_4 and $[PdCl(PPh_3)_3]BF_4$ (162 MHz, CD_2Cl_2).

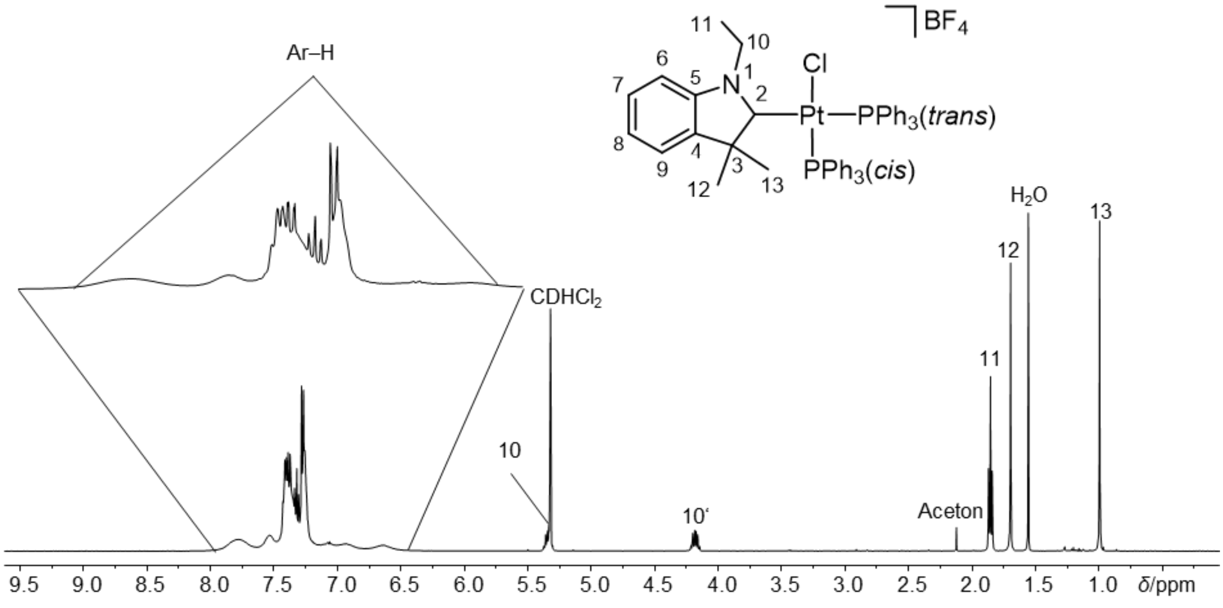

Figure S9. ^{1}H NMR spectrum of *cis*-[**4**]BF_4 (400 MHz, CD_2Cl_2).

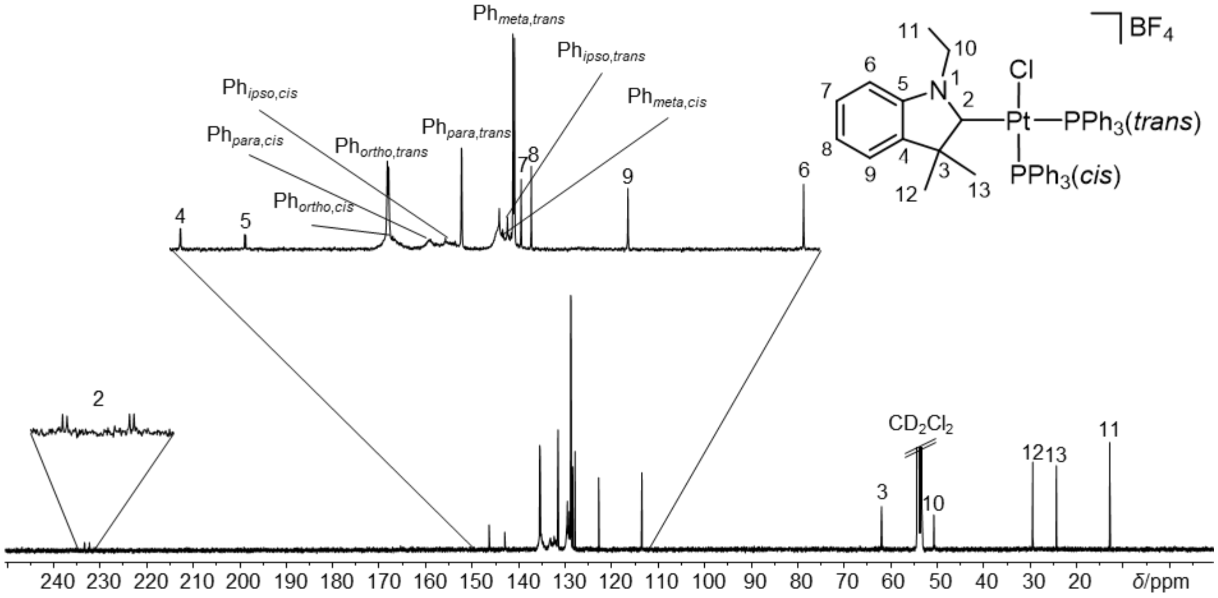

Figure S10. ^{13}C NMR spectrum of *cis*-[**4**]BF_4 (101 MHz, CD_2Cl_2).

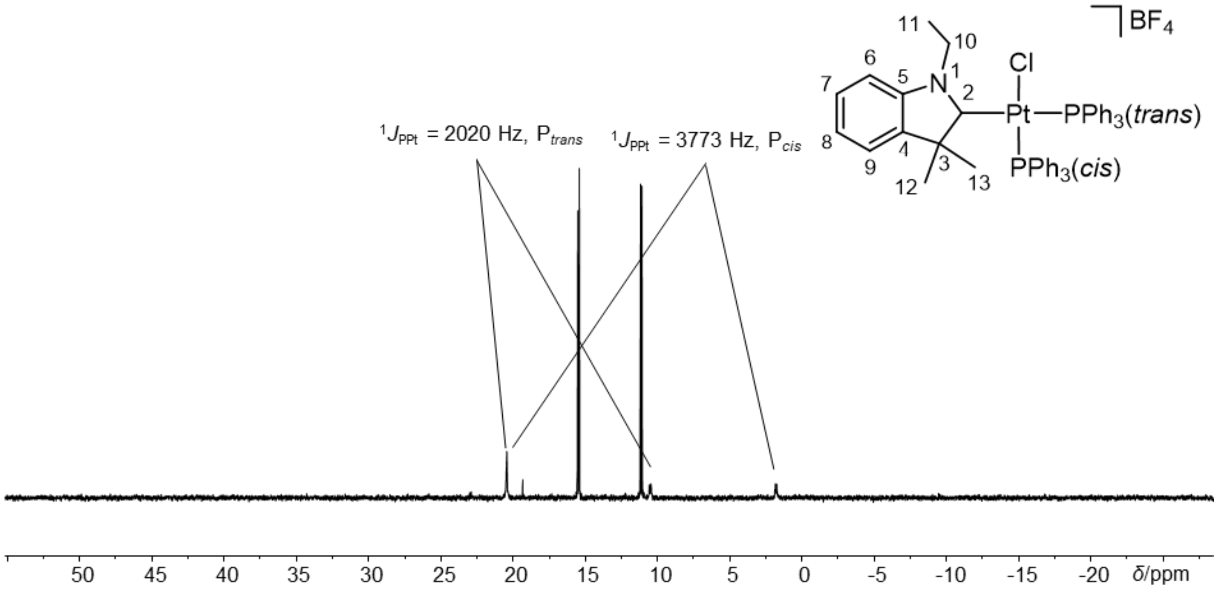

Figure S11. ^{31}P NMR spectrum of *cis*-[**4**]BF_4 (162 MHz, CD_2Cl_2).

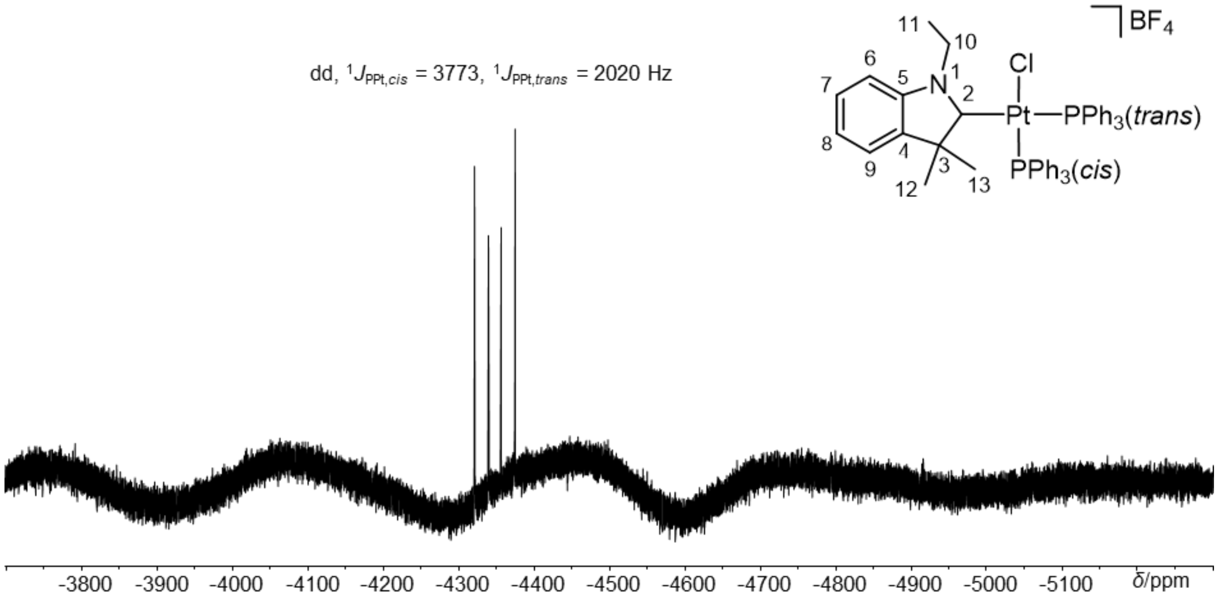

Figure S12. ^{195}Pt NMR spectrum of *cis*-[**4**]BF_4 (107 MHz, CD_2Cl_2).

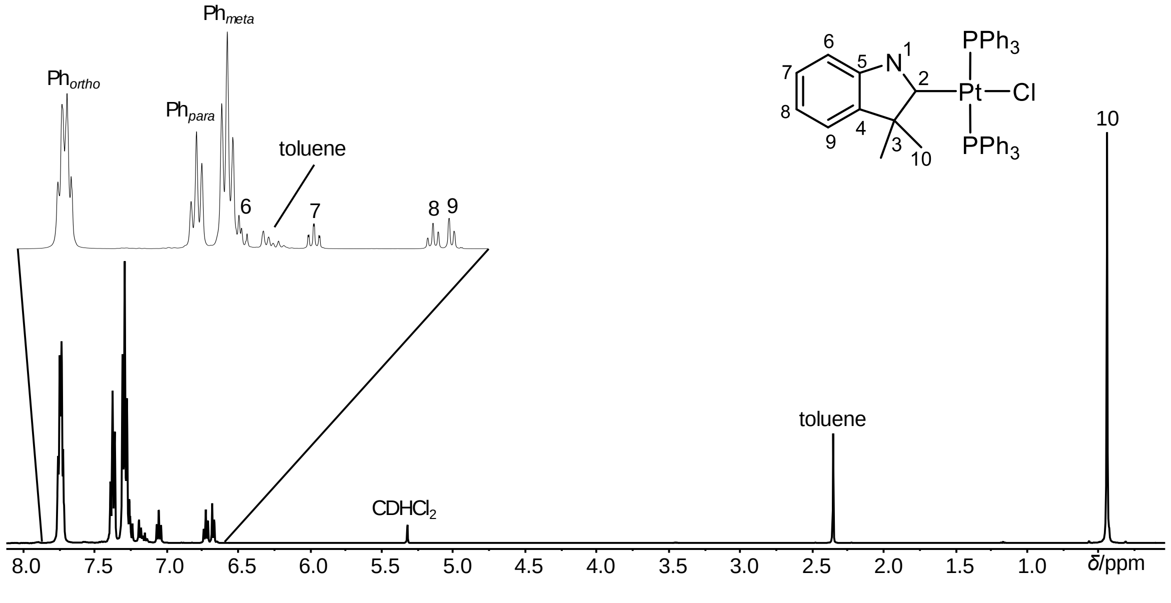

Figure S13. ^{1}H NMR spectrum of *trans*-[**5**] (400 MHz, CD_2Cl_2).

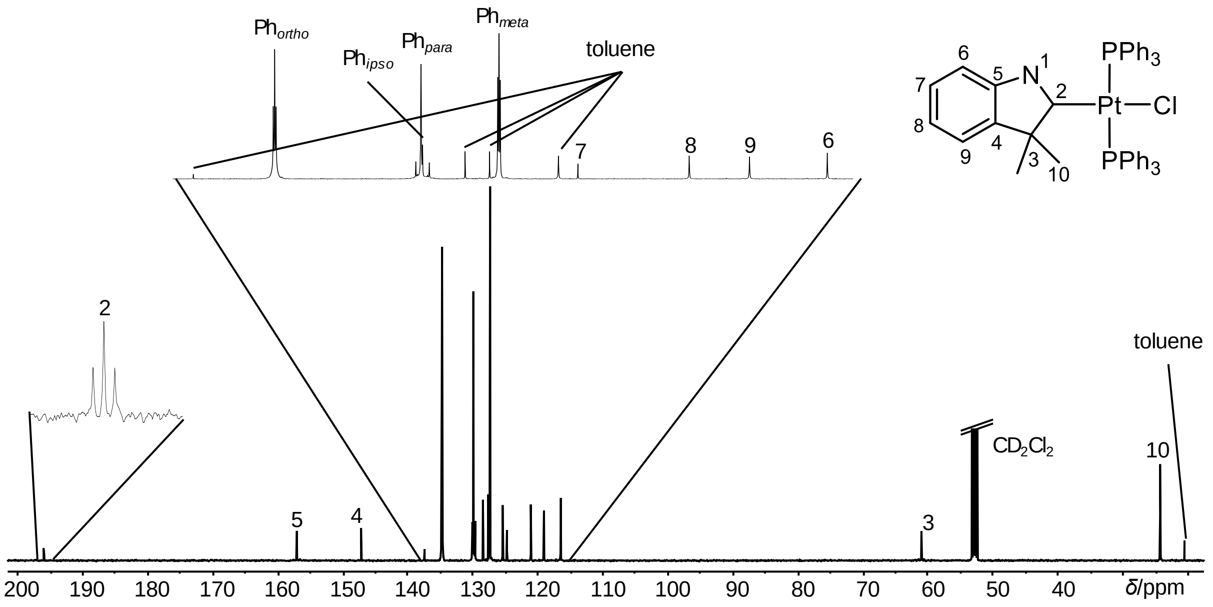

Figure S14. ^{13}C NMR spectrum of *trans*-[**5**] (101 MHz, CD_2Cl_2).

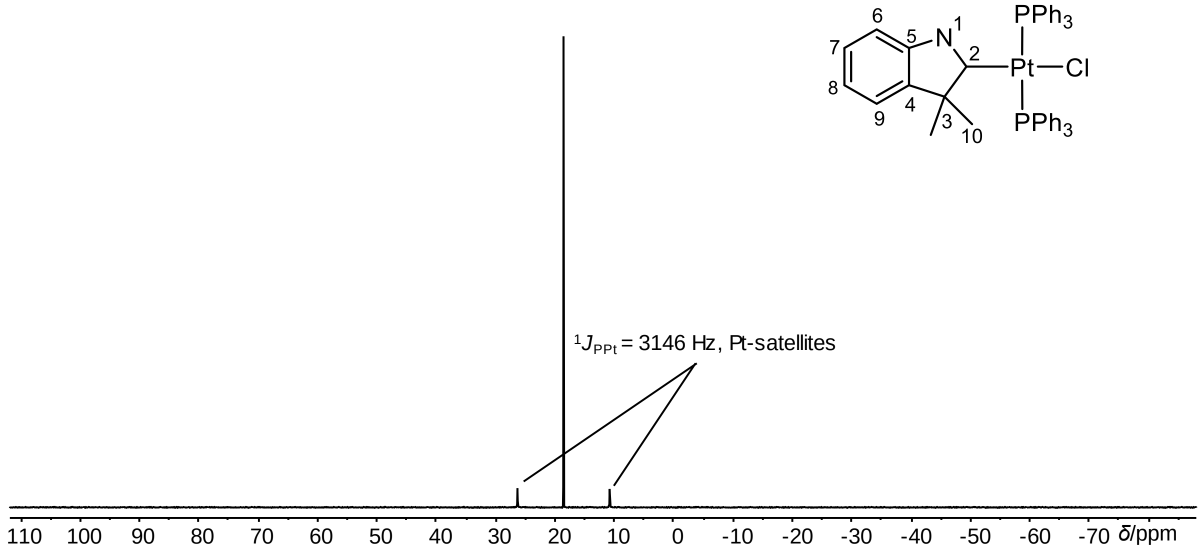

Figure S15. ^{31}P NMR spectrum of *trans*-[**5**] (162 MHz, CD_2Cl_2).

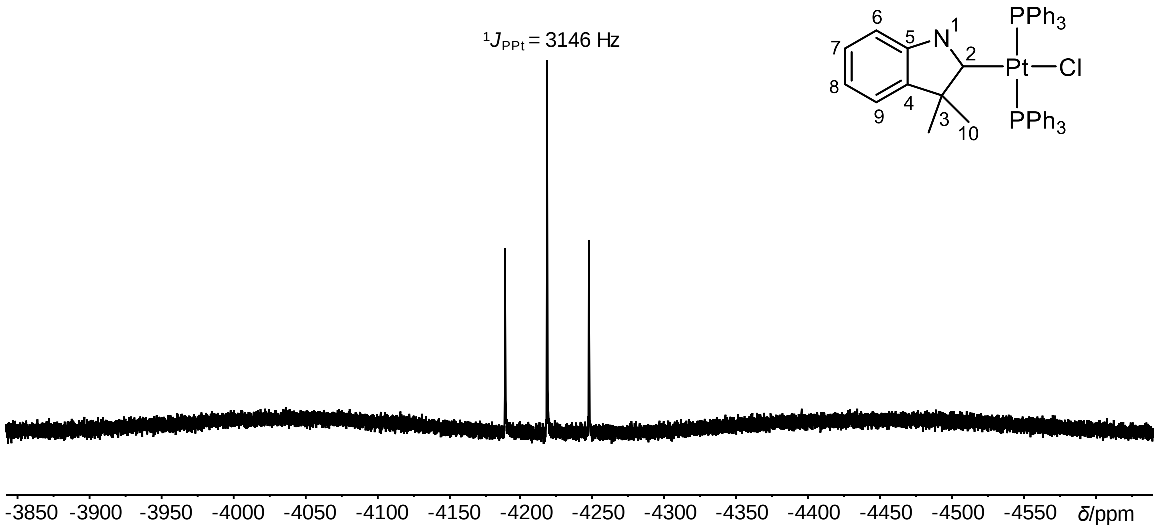

Figure S16. ^{195}Pt NMR spectrum of *trans*-[**5**] (107 MHz, CD_2Cl_2).

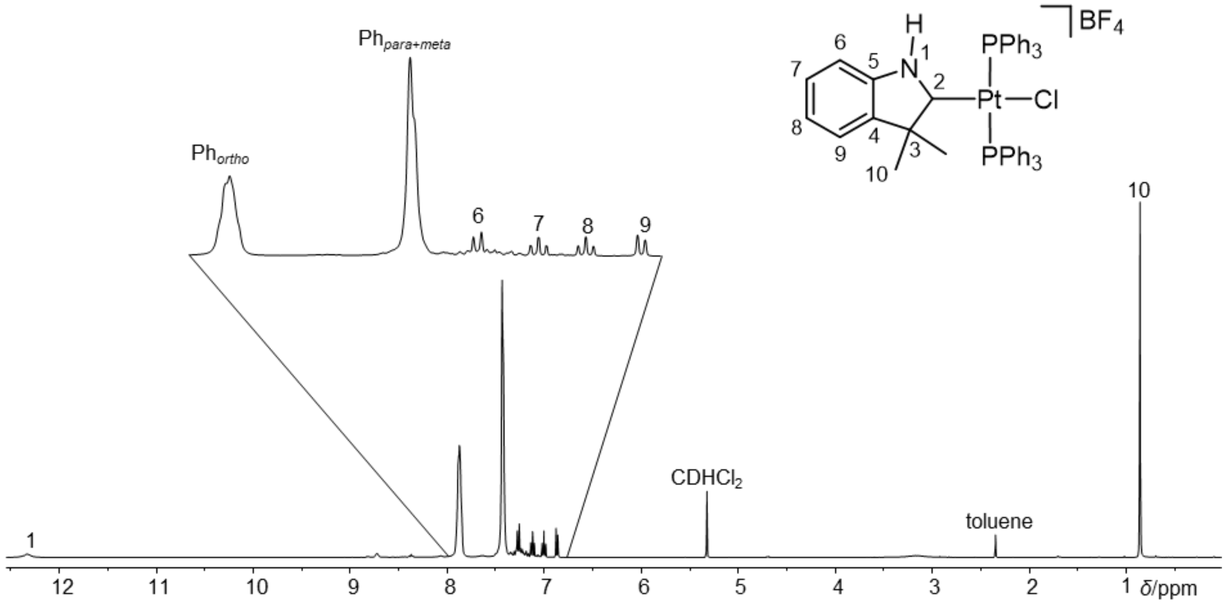

Figure S17. ^{1}H NMR spectrum of *trans*-[**6**]BF_4 (400 MHz, CD_2Cl_2).

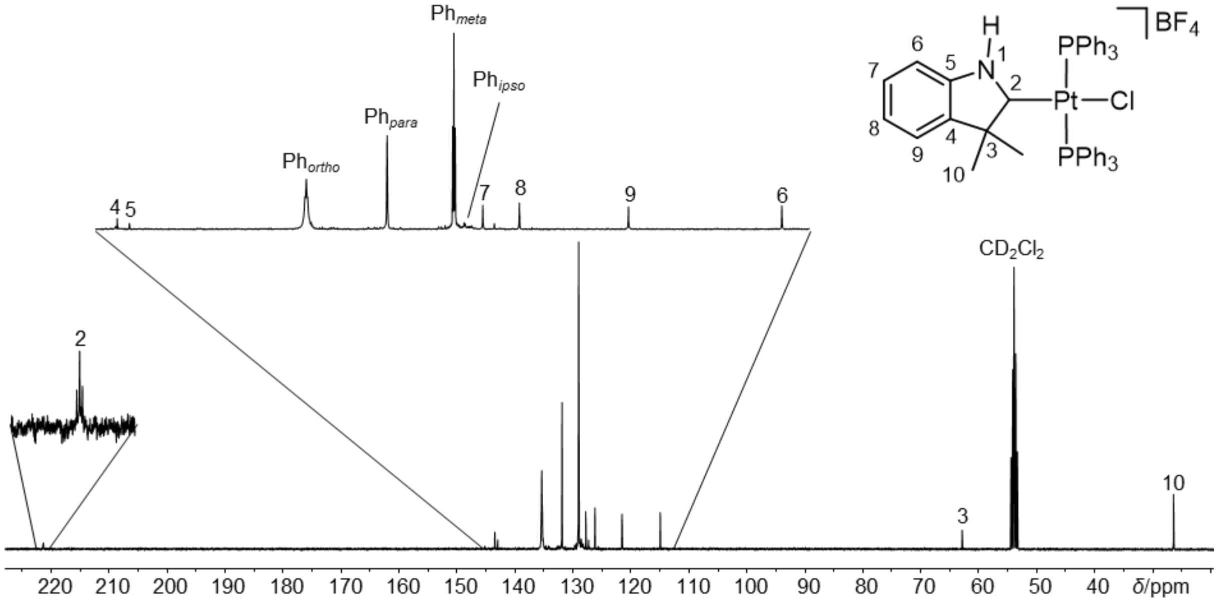

Figure S18. ^{13}C NMR spectrum of *trans*-[**6**]BF$_4$ (101 MHz, CD$_2$Cl$_2$).

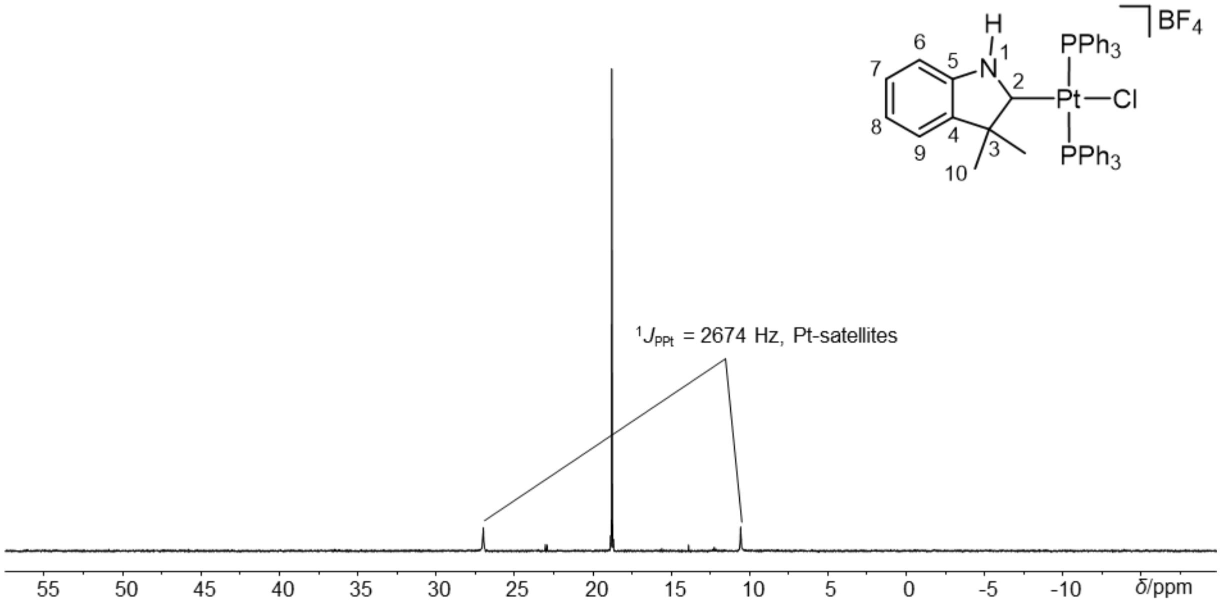

Figure S19. ^{31}P NMR spectrum of *trans*-[**6**]BF$_4$ (162 MHz, CD$_2$Cl$_2$).

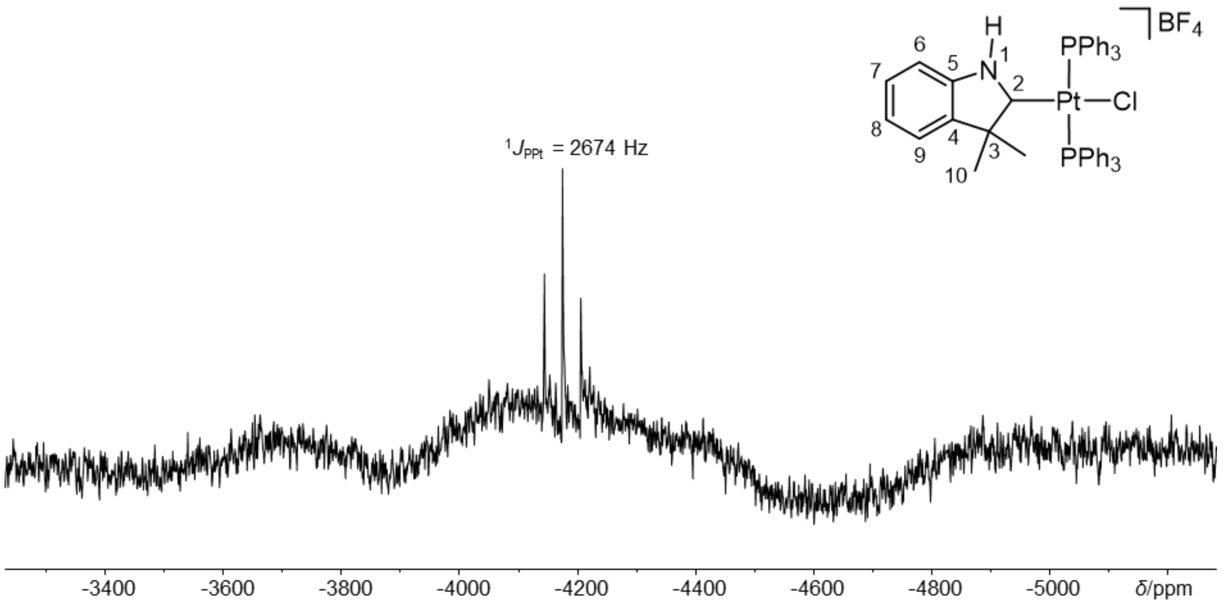

Figure S20. ^{195}Pt NMR spectrum of trans-[**6**]BF_4 (107 MHz, CD_2Cl_2).

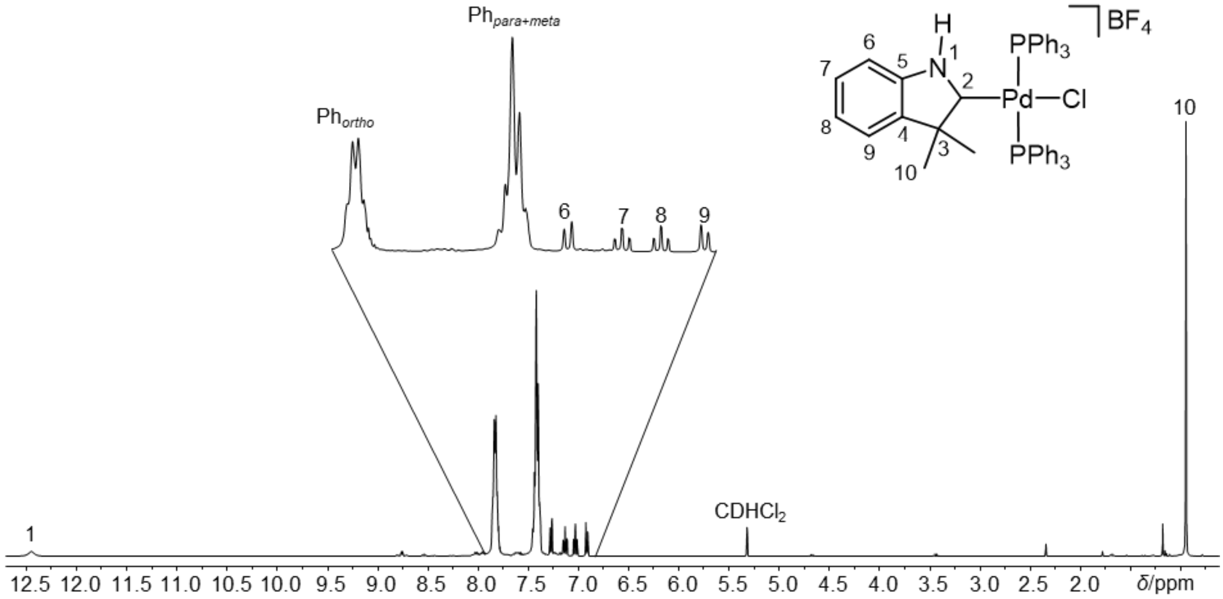

Figure S21. ^{1}H NMR spectrum of *trans*-[**7**]BF_4 (400 MHz, CD_2Cl_2).

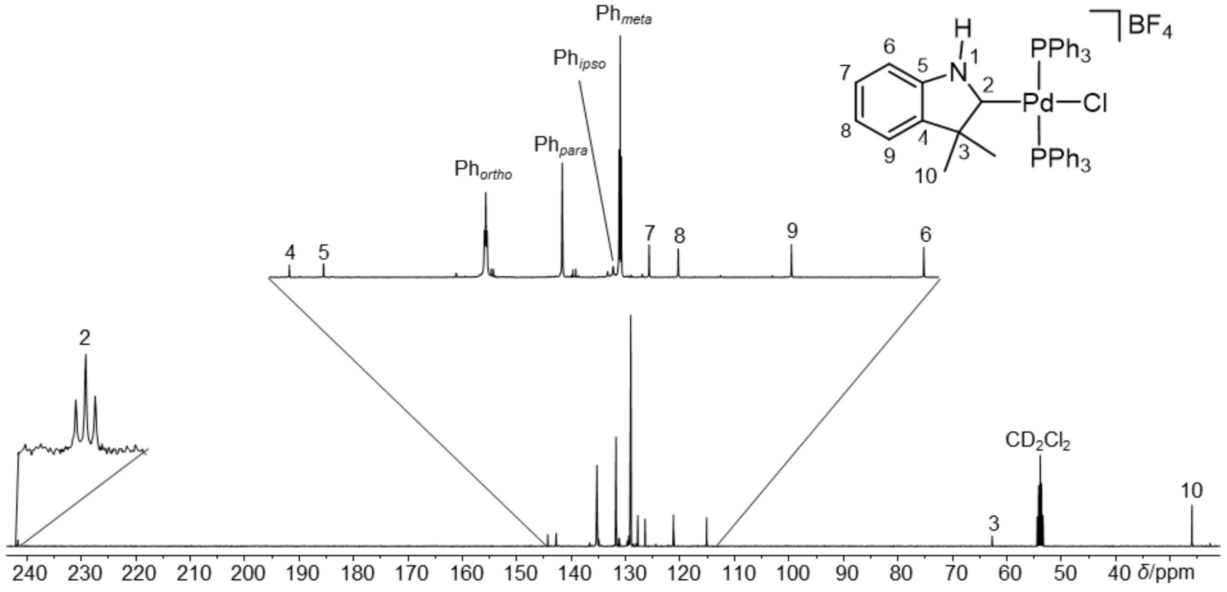

Figure S22. $^{13}C\{^1H\}$ NMR spectrum of *trans*-[**7**]BF_4 (101 MHz, 300 K, CD_2Cl_2).

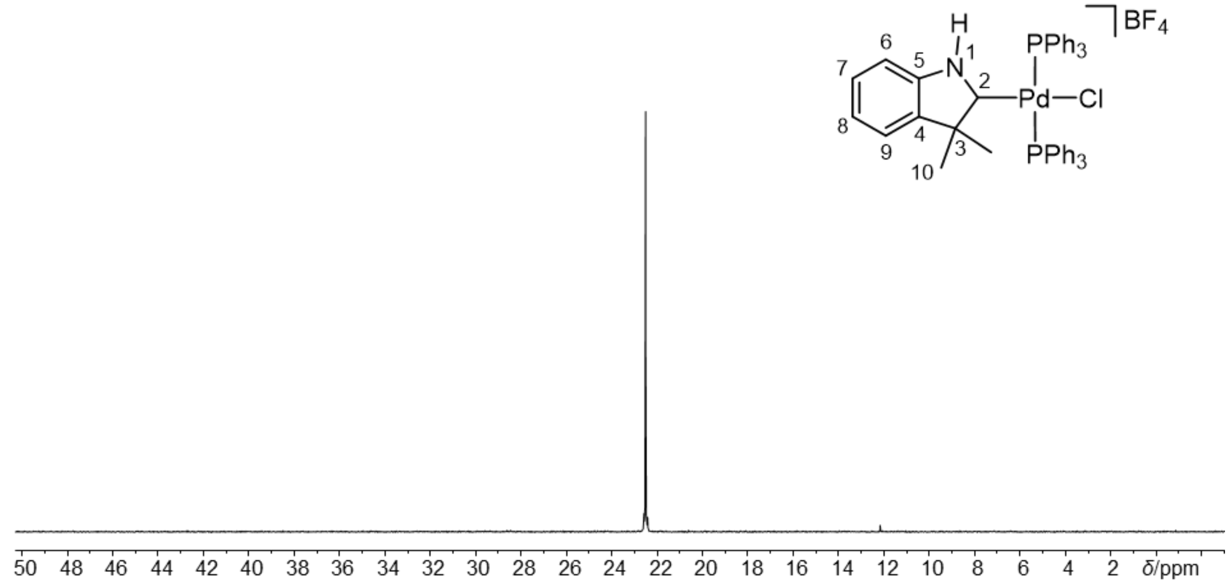

Figure S23. ^{31}P NMR spectrum of *trans*-[**7**]BF_4 (162 MHz, CD_2Cl_2).

10. References

S1. Robertson, D. W.; Krushinski, J. H.; Kau, D. Synthesis of ^{14}C- and ^{2}H-Labeled 1,3-Dihydro-3,3-dimethyl-5-(1,4,5,6-tetrahydro-6-oxo-3-pyridazionyl)-2*H*-indol-2-one (LY195115), an Orally

Effective Positive Ionotrope. *J. Label. Compd. Radiopharm.* **1986**, *23*, 343–354.

S2. Hünig, S.; Balli, H. Azofarbstoffe durch oxidative Kupplung. *J. Liebigs Ann. Chem.* **1957**, *609*, 160–172.

S3. Krause, L.; Herbst-Irmer, R.; Sheldrick, G. M.; Stalke, D. Comparison of silver and molybdenum microfocus X-ray sources for single-crystal structure determination. *J. Appl. Crystallogr.* **2015**, *48*, 3–10.

S4. Blessing, R. H. An Empirical Correction for Absorption Anisotropy. *Acta Cryst.* **1995**, *A51*, 33–38.

S5. Sheldrick, G. M. *SHELXT* – Integrated space-group and crystal-structure determination. *Acta Cryst.* **2015**, *A71*, 3–8.

S6. Sheldrick, G. M. Crystal structure refinement with *SHELXL*. *Acta Cryst.* **2015**, *C71*, 3–8.
